# Unveiling the Gating Mechanism of CRAC Channel: A Computational Study

**DOI:** 10.3389/fmolb.2021.773388

**Published:** 2021-12-14

**Authors:** Carlo Guardiani, Delia Sun, Alberto Giacomello

**Affiliations:** Dipartimento di Ingegneria Meccanica e Aerospaziale, Sapienza Università di Roma, Rome, Italy

**Keywords:** CRAC channel, gating mechanism, hydrophobic gating, allosteric gating, molecular dynamics, targeted molecular dynamics, pore helix rotation, structural instability of P288L

## Abstract

CRAC channel is ubiquitous and its importance in the regulation of the immune system is testified by the severe immunodeficiencies caused by its mutations. In this work we took advantage of the availability of open and closed structures of this channel to run for the first time simulations of the whole gating process reaching the relevant time-scale with an enhanced sampling technique, Targeted Molecular Dynamics. Our simulations highlighted a complex allosteric propagation of the conformational change from peripheral helices, where the activator STIM1 binds, to the central pore helices. In agreement with mutagenesis data, our simulations revealed the key role of residue H206 whose displacement creates an empty space behind the hydrophobic region of the pore, thus releasing a steric brake and allowing the opening of the channel. Conversely, the process of pore closing culminates with the formation of a bubble that occludes the pore even in the absence of steric block. This mechanism, known as “hydrophobic gating”, has been observed in an increasing number of biological ion channels and also in artificial nanopores. Our study therefore shows promise not only to better understand the molecular origin of diseases caused by disrupted calcium signaling, but also to clarify the mode of action of hydrophobically gated ion channels, possibly even suggesting strategies for the biomimetic design of synthetic nanopores.

## 1 Introduction

The ubiquitous Calcium Release Activated Calcium channel (CRAC) mediates Ca^2+^-influx through the plasma membrane of non-excitable cells in metazoans activating communication cascades that elicit a wide range of functions (Prakriya and Lewis, 2015) like gene expression, cell proliferation, secretion of inflammatory mediators, and cell migration. The importance of CRAC channel is highlighted by the pathological effects of both gain of function and loss of function mutations (Lacruz and Feske, 2015). While loss of function mutations cause the severe combined immunodeficiency (SCID)-like disease, autoimmunity, muscular hypotonia, and ectodermal dysplasia, gain of function mutations have been linked to non-syndromic tubular aggregate myopathy and York platelet and Stormorken syndromes. Although structures of the closed and open states are available (Hou et al., 2012, 2018; Liu et al., 2019) and despite a significant body of experimental research (Yamashita et al., 2017; Yeung et al., 2018), the gating mechanism of this channel is still a matter of debate, a central issue being the allosteric propagation (Zhou et al., 2019; Yeung et al., 2020) of the signal from the activator binding site to the central pore helices.

The CRAC channel mediates the store operated calcium entry (SOCE) (Prakriya and Lewis, 2015), a process of calcium influx triggered by the depletion of the calcium stores of the endoplasmic reticulum (ER). When ER calcium stores are depleted, calcium dissociates from the calcium-sensing, luminal EF-hand domain of STIM1, a single pass protein of the ER membrane. STIM1 then migrates to the junctions between ER and plasma membranes where it oligomerizes and undergoes a conformational transition that exposes the CRAC activation domain (CAD) (Park et al., 2009). The interaction of STIM1 CAD domain with CRAC outermost helices (TM4) induces the opening of the CRAC channel that results in an influx of calcium that refills the stores of the ER and activates a number of signalling pathways including those necessary for the activation of immune response genes in T cells (Feske et al., 2005).

There are three human isoforms of CRAC encoded by genes Orai1, 2, and 3 (Prakriya and Lewis, 2015). Since an experimental structure of the human CRAC channel is not yet available, most computational works (Dong et al., 2013, 2014; Yamashita et al., 2017) (including ours) have been performed on the *Drosophila melanogaster* protein encoded by gene Orai that shares 73% sequence identity with Orai1. From a structural point of view, CRAC is a homo-hexamer (Hou et al., 2012) with each of the six identical subunits formed by four trans-membrane helices. CRAC helices are arranged in three concentric layers. Helix TM1 forms the innermost layer that lines the pore, helices TM2 and TM3 form the central layer and helix TM4 forms the outermost layer. The pore wall of this channel exhibits an extremely complex composition. Close to the extra-cellular mouth E178 forms a negatively charged glutamate ring that imparts cation selectivity and thus acts as a Selectivity Filter. The central part of the pore is occupied by a hydrophobic region with three rings of hydrophobic residues (L167, F171 and V174). Finally, close to the cytosolic mouth we find a basic region with three rings of positively charged residues (R155, K159 and K163).

**FIGURE 1:**
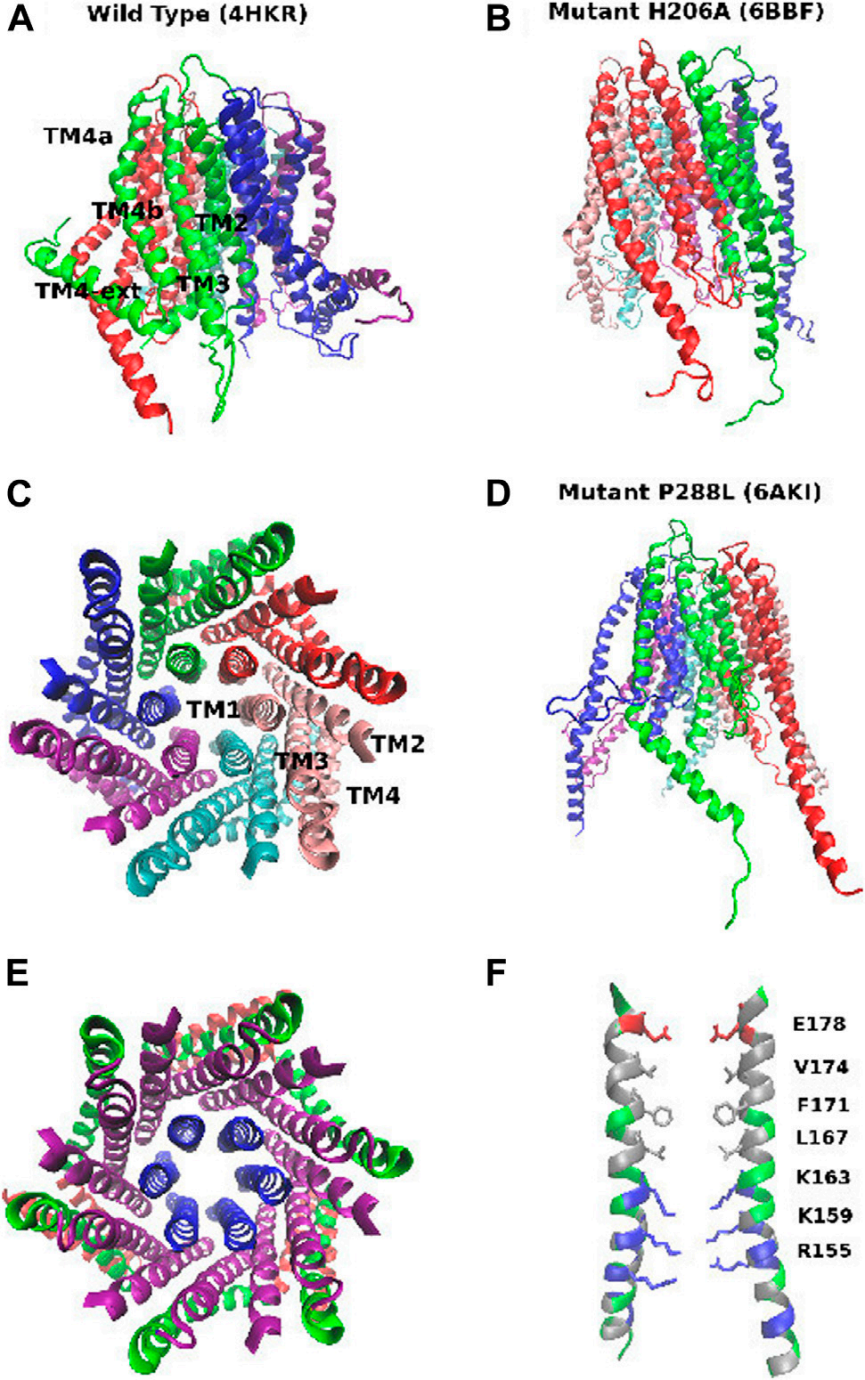
Structure of the CRAC channel. Panel **(A)**: structure of the closed state (PDB: 4HKR): side view. Panel **(B)**: structure of the constitutively open mutant H206A (PDB: 6BBF). Panel **(C)**: structure of the closed state (PDB: 4HKR): top view, with the six subunits shown in different colors. Panel **(D)**: structure of mutant P288L (PDB: 6AKI). Panel **(E)**: structure of the closed state (PDB: 4HKR): top view highlighting the concentric arrangement of the 3 rings of helices. Helices TM1: blue, helices TM2 and TM3: purple, helices TM4a and TM4b: green, helices TM4-ext: red. Panel **(F)**: structure of the pore of the closed state. Hydrophobic residues are shown in silver, positively charged residues in blue, negatively charged residues in red, neutral but polar residues in green. Residues of the E-ring, hydrophobic region and basic region are shown in a stick representation.

Three main experimental structures of CRAC are currently available (Figure 1). The structure with PDB code 4HKR (Hou et al., 2012) corresponds to the closed state. The structure is characterized by a narrow pore and a double bent TM4 helix that is thus split into three helical segments: TM4a on the extracellular side, the central TM4b, and the TM4-ext helix on the cytosolic side. The TM4-ext helices of neighbouring subunits associate with one another to form a coiled-coil structure that is believed to be the docking point of the STIM1 activator. Besides the closed state, recently the structures of two constitutively open mutants have been disclosed, P288L (PDB: 6AKI) (Liu et al., 2019) and H206A (PDB: 6BBF) (Hou et al., 2018). While 6BBF is characterized by a wide pore and fully extended TM4 helices, 6AKI shows an arrangement of the pore helices similar to that of the closed state (4HKR) with partially bent TM4 helices.

**FIGURE 2:**
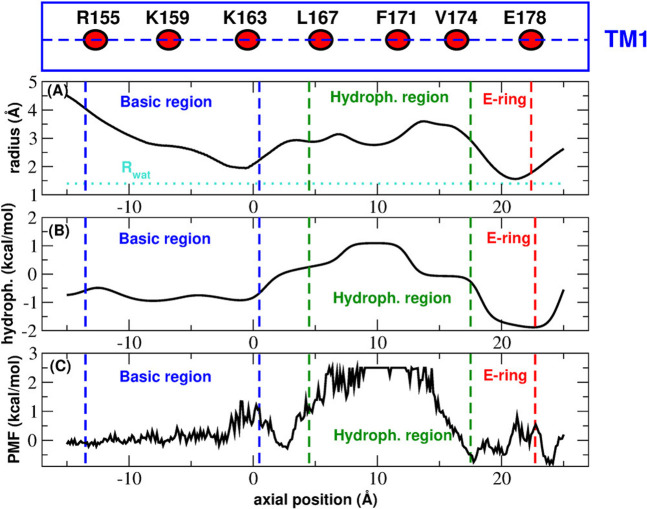
Functional state assignment of CRAC structure 4HKR. Panel **(A)**: average radius profile during the equilibrim simulation. The dotted line corresponds to the radius of a water molecule. Panel **(B)**: hydrophobicity profile. In the Wimley-White scale positive and negative hydrophobicity values correspond to hydrophobic and hydrophylic amino acids respectively. Panel **(C)**: Potential of Mean Force of water permeation as a function of the position along the axis of the pore. The blue and green dashed lines mark the boundaries of the basic and hydrophobic region respectively. The red dashed lines shows the position of the glutamate ring. The box above Panel **(A)** is a cartoon representation of helix TM1 showing the position of the key residues (average position of the center of mass of C_*α*_ atoms) along the helix axis (dashed line).

Many aspects of CRAC channel need to be clarified. For instance the assignment of structure 4HKR to the closed state based on experimental calcium flow measurements, is at odds with a pore radius (Figure 2) that is everywhere large enough to accommodate a water molecule or a dehydrated calcium ion. The functional occlusion coexisting with a geometric permeability of the pore suggests a *hydrophobic gating* mechanism (Aryal et al., 2015) i.e*.* a phenomenon of evaporation in nanoconfinement whereby the formation of a vapour bubble stops the flow of liquid water and ions. Indeed, the hydrophobic region of the pore would seem ideally suited for this kind of mechanism. Many computational studies have monitored the amount of water in CRAC pore and, even if there is a consensus that the water content of the hydrophobic region in the closed state is very low, the results still show a significant variability. Yeung et al. (2018), for instance, show a trajectory where the bubble appears and disappears intermittently for the first ∼100 ns but the pore is fully wetted for the remaining ∼250 ns. In the work by Dong et al., (2013), on the other hand, a proper bubble never forms and, even in the closed state, the hydrophobic region is always crossed by a number of thin chains of water molecules. These results are at odds with those reported by Frischauf et al. (2019). These authors simulated a homology model of human CRAC discovering that in the wild type the hydrophobic region is always fully dewetted.

Another controversial issue regards the gating mechanism. In an early paper Yamashita et al. (2017) proposed a mechanism based on a rotation of pore helices aimed at displacing the bulky side chains of hydrophobic residues occluding the hydrophobic region. The rotation of pore helices, however, was not confirmed by the simulations of Frischauf et al. (2019), neither was it observed in the crystal structures of P288L (Liu et al., 2019) and H206A (Hou et al., 2018) mutants.

The major unresolved question about CRAC channel, however, concerns the molecular mechanism by which the STIM1 gating signal is communicated at large distance to the pore region (Zhou et al., 2019; Yeung et al., 2020). The existence of a highly cooperative and strongly allosteric gating mechanism is supported by the discovery (Nesin et al., 2014; Boehm et al., 2017; Garibaldi et al., 2017) of an increasing number of pathologic mutations located far from the pore region. This evidence suggests that the transmembrane domains of CRAC channel may be involved in the process of propagation of the wave of conformational change that originates at the STIM1 binding site on helices TM4 and heads to the hydrophobic region of the pore helices where the gating motion occurs. Within this framework, the gating mechanism would not just require a localized motion of the pore helices, but a globally concerted motion involving the whole protein. A significant work on this issue has been performed by Yeung et al. (2018) who performed scanning mutagenesis to identify a number of residues in transmembrane domains whose mutation leads to constitutive activation of the pore. In particular, they identified the role of *steric brake* of H206 and they highlighted the importance of a cluster of hydrophobic residues at the TM1-TM2/3 interface.

In this work we took advantage of the availability of closed (Hou et al., 2012) and open state (Hou et al., 2018; Liu et al., 2019) structures to simulate for the first time the conformational transition from the closed to the open state and *vice versa*. Since this process is likely to occur at least on the milli-second time-scale, it cannot be simulated with equilibrium MD techniques. We thus resorted to Targeted MD (Schlitter et al., 1994), an enhanced sampling technique that steers the system between given end states applying a biasing potential to the RMSD between the current and target conformations. Since its introduction, a number of successfull applications have shown that TMD can yield qualitatively correct pathways of conformational changes. As an example, TMD was used to study the activation pathway of *β*2 adrenergic receptor (Xiao et al., 2015), the allosteric mechanism of calmodulin opening (Liang et al., 2017), and the role of the cytoplasmic domain in the gating of KcsA channel (Li et al., 2013). Before running TMD, we ran equilibrium simulations of the available closed and open strauctures. The spontaneous formation of a bubble occluding the pore of the closed state in the absence of steric block, highlighted a mechanism of hydrophobic gating (Aryal et al., 2015). The simulation of the putatively open mutants H206A and P288L showed for the first time that the former was structurally stable while the latter underwent a spontaneous bending of helices TM4. This led us to suggest that P288L is more likely an intermediate in the gating process than the open state. As a result we chose the H206A structure as the open state to use in TMD simulations. In agreement with Yamashita et al. (2017), the TMD simulations showed rotation of pore helices contributing to the change of pore radius even if an important role also appears to be played by TM1 helix displacement. The analysis of TMD simulations highlighted two important events in the gating mechanism: a coordinated motion of helices TM1, TM3, and TM4b that leads to the opening of the basic region of the pore and a displacement of the side chain of H206 creating an empty space behind the hydrophobic region of helices TM1. This mechanism appears to be consistent with the steric brake model put forward by Yeung et al. (2018).

The paper is organized as follows. In Section 2.1 and Section 2.2 we present the equilibrium simulations of the closed and putatively open states respectively. In Section 2.3 we analyze TMD simulations first focusing on the rotation of pore helices (Section 2.3.1). We then discuss the results of the contact analysis (Section 2.3.2) and the analysis of inter-helical distances (Section 2.3.3) that highlight important details of the gating mechanism. Finally, Section 3 is devoted do the discussion, while Section 4 illustrates the methods employed in our calculations.

## 2 Results

### 2.1 Equilibrium Simulation of the Closed State

Our analysis of the CRAC channel began with the equilibrium simulation of the closed state (PDB ID: 4HKR). The functional state of an ion channel is customarily predicted (Rao et al., 2019) on the grounds of the radius profile. Supplementary Figure S1 shows the radius profile of the crystal structure and the profile of the radius averaged along the equilibrium simulations. Both profiles are everywhere larger than the radius of a single water molecule showing the absence of steric obstruction. From a purely geometric point of view the channel is thus expected to be water-permeable. This prediction is confirmed by the space filling representation of the pore shown in Supplementary Figure S2. According to the color code employed by the HOLE program (Smart et al., 1996), green regions are wide enough to accommodate no more than a single water molecule, while blue regions can accommodate two or more water molecules. Supplementary Figure S2 shows a bottleneck at the level of the Glu-ring and other two at the level of the basic region. In these points, the side chains project towards the center of the pore reducing its radius but there is still enough space for a single chain of water molecules. Except for these three points the pore can everywhere be occupied by two or more water molecules.

This analysis is inconsistent with the electrophysiological characterization of this structure (Hou et al., 2012) clearly showing it corresponds to the closed state. This inconsistency can be explained if the radius profile is compared with the hydrophobicity profile and the Potential of Mean Force of water permeation (Figure 2). Figure 2 shows that, even if in principle the pore is wide enough to host one or more water molecules (Figure 2A), the chemistry of the wall determines a peak of the hydrophobicity profile (Figure 2B) corresponding to the hydrophobic region of the pore. The hydrophobicity peak also corresponds to the maximum of the Potential of Mean Force for water permeation (Figure 2C). Taken together, these results suggest that the pore is functionally occluded even in the absence of steric block, which is the signature of *hydrophobic gating*.

**FIGURE 3:**
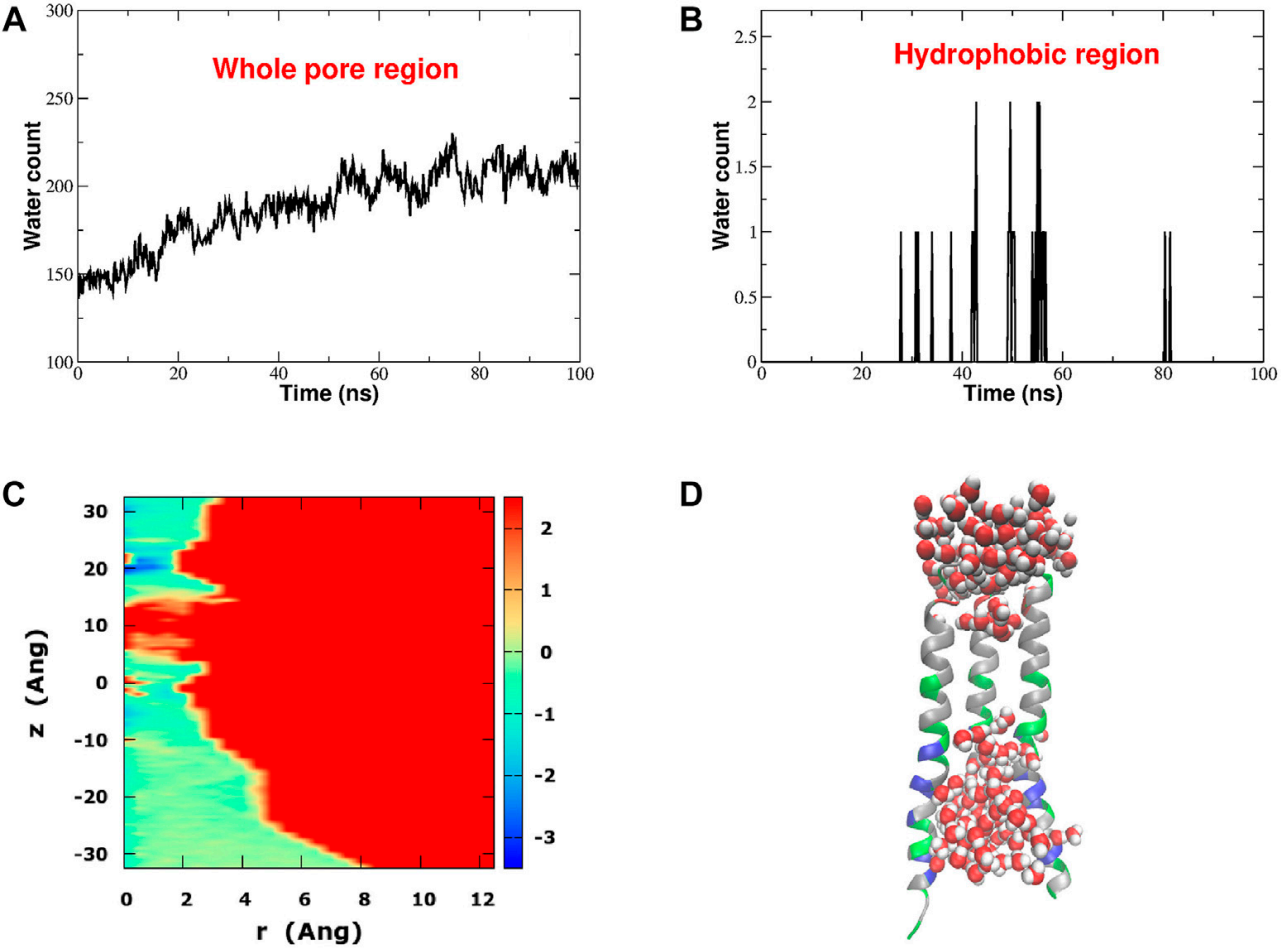
Hydrophobic gating in CRAC channel. Panel **(A)**: time evolution of the number of water molecules in the whole pore region. Panel **(B)**: time evolution of the number of water molecules in the hydrophobic region. Panel **(C)**: Potential of Mean Force of water (in kcal/mol) as a function of the distance *r* from, and the position *z* along the pore axis. Panel **(D)**: distribution of water in the pore region in the last frame of the 100 ns equilibrium simulation. For the sake of graphical clarity only three of the six pore helices are shown. Pore helices are colored according to residue type: positively charged residues are blue, negatively charged residues are red, polar but neutral residues are green and hydrophobic residues are white.

Hydrophobic gating is a phenomenon of evaporation in conditions of nanoconfinement (Roth et al., 2008). If the water-wall interactions are weaker than water-water interactions, a bubble may form, preventing the flow of liquid water and ions. This pattern is clearly manifested in our equilibrium simulation. Figure 3 shows the time evolution of the number of water molecules in the whole pore region (Figure 3A) and in the hydrophobic region (Figure 3B). The water count in the whole pore slightly increases during the first half of the simulation and eventually it stabilizes around an average value of 200 water molecules. Conversely, if we restrict our attention to the hydrophobic region, this district appears to be almost always devoid of water safe for a few occasional intrusions of 1 or 2 water molecules at the boundary with the Glu-ring and the basic region. Indeed Figure 3D, corresponding to the final frame of the simulation, shows that the hydrophobic region of the pore is dry. The bubble appears early, already during the constrained stage of the equilibration, and it is stably maintained throughout the whole simulation of 100 ns. As a result, the radial-axial PMF of water permeation reported in Figure 3C, highlights a high free energy zone (in red) in the hydrophobic region where water has very little likelihood to reside.

### 2.2 Equilibrium Simulation of Putatively Open States

The main purpose of this work is to study the gating transition steering the CRAC channel from the open to the closed state and back using Targeted Molecular Dynamics simulations (Schlitter et al., 1994). Since this technique requires knowledge of the end states of the transition, it is extremely important to computationally characterize the recently resolved structures of two putatively open states, mutants H206A (PDB ID: 6BBF) (Hou et al., 2018) and P288L (PDB ID: 6AKI) (Liu et al., 2019). This analysis will allow us to choose the most appropriate conformation to use as the open state in TMD simulations.

**FIGURE 4:**
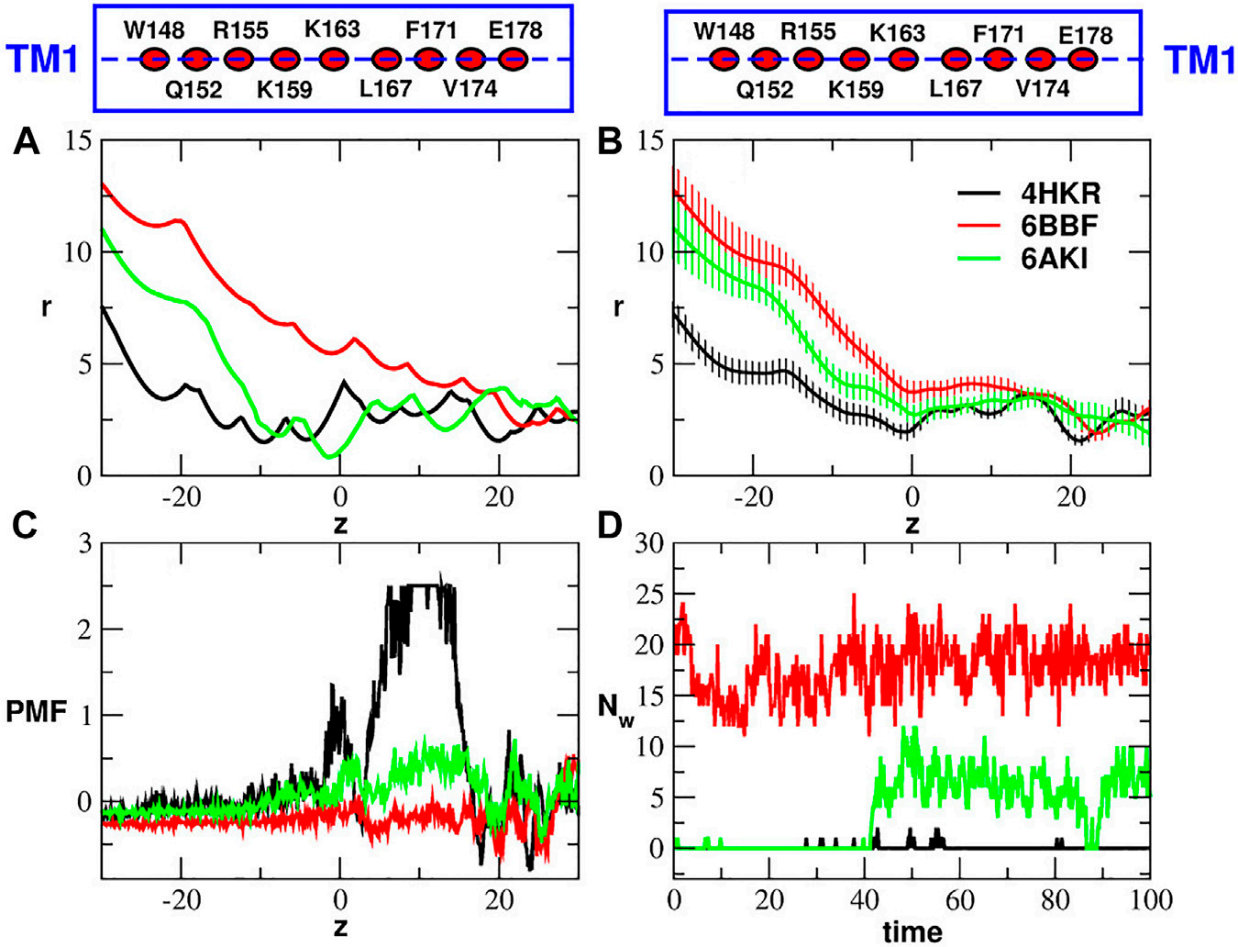
Comparison of closed and putatively open structures. Panel **(A)**: radius profiles of the crystal structure of the wild type (4HKR) and the two putatively open structures (6BBF and 6AKI). Panel **(B)**: average radius profiles computed during the equilibrium simulations of 4HKR, 6BBF and 6AKI. The error is quantified by the standard deviation of the radius. In panels **(A)** and **(B)** the pore radius *r* and the position along the pore axis *z* are both expressed in Å. Panel **(C)**: Potential of Mean Force of water (in kcal/mol) as a function of the axial position *z* (in Å). Panel **(D)**: number of water molecules *N*_*w*_ in the hydrophobic region as a function of simulation time (in ns). The boxes above Panels **(A)** and **(B)** are cartoon representations of helix TM1 showing the position of the key residues (average position of the center of mass of C_*α*_ atoms) along the helix axis (dashed lines).

Figure 4 shows the radius profile of the crystal structures of the closed and open states (Figure 4A) as well as the radius profile averaged during the equilibrium simulation of the three systems (Figure 4B). The radius profiles of the experimental structures are very similar at the level of the Glu-ring, but in the hydrophobic and basic regions the pore of 6BBF appears to be much larger than that of the other two structures. Interestingly enough, even if 6AKI was predicted (Liu et al., 2019) to represent the open state, its radius profile is much more similar to that of the closed state (4HKR) than to that of the other putatively open state (6BBF). During the equilibrium simulation, the radius profile of 6AKI tended to drift towards that of 6BBF until it settled on a curve intermediate between those of the closed state (4HKR) and of the other putatively open state (6BBF). As shown in Figure 4C, the different pore geometries affect the Potential of Mean Force of water permeation. As expected, the closed state is characterized by a high free energy barrier at the level of the hydrophobic region where the vacuum bubble prevents the flow of liquid water. Conversely, during the equilibrium simulation the wide pore of 6BBF is completely water filled. Since water density inside the pore is comparable to that in the bulk, the PMF profile is almost flat. Finally, the PMF profile of 6AKI also shows a barrier, smaller than that of the closed state but higher than what expected for an open state. The origin of this barrier can be understood monitoring the number of water molecules in the hydrophobic region of the three systems (Figure 4D). The plot clearly shows that, during the first 40 ns of the simulation, the hydrophobic region of 6AKI is also occupied by a bubble, which, during the second half of the trajectory, becomes water-filled. The process is also shown in Supplementary Movie SM1. This event, similar to a spontaneous transition from the closed to the open state, suggests the opportunity of a deeper investigation on the structural stability of 6AKI.

**FIGURE 5:**
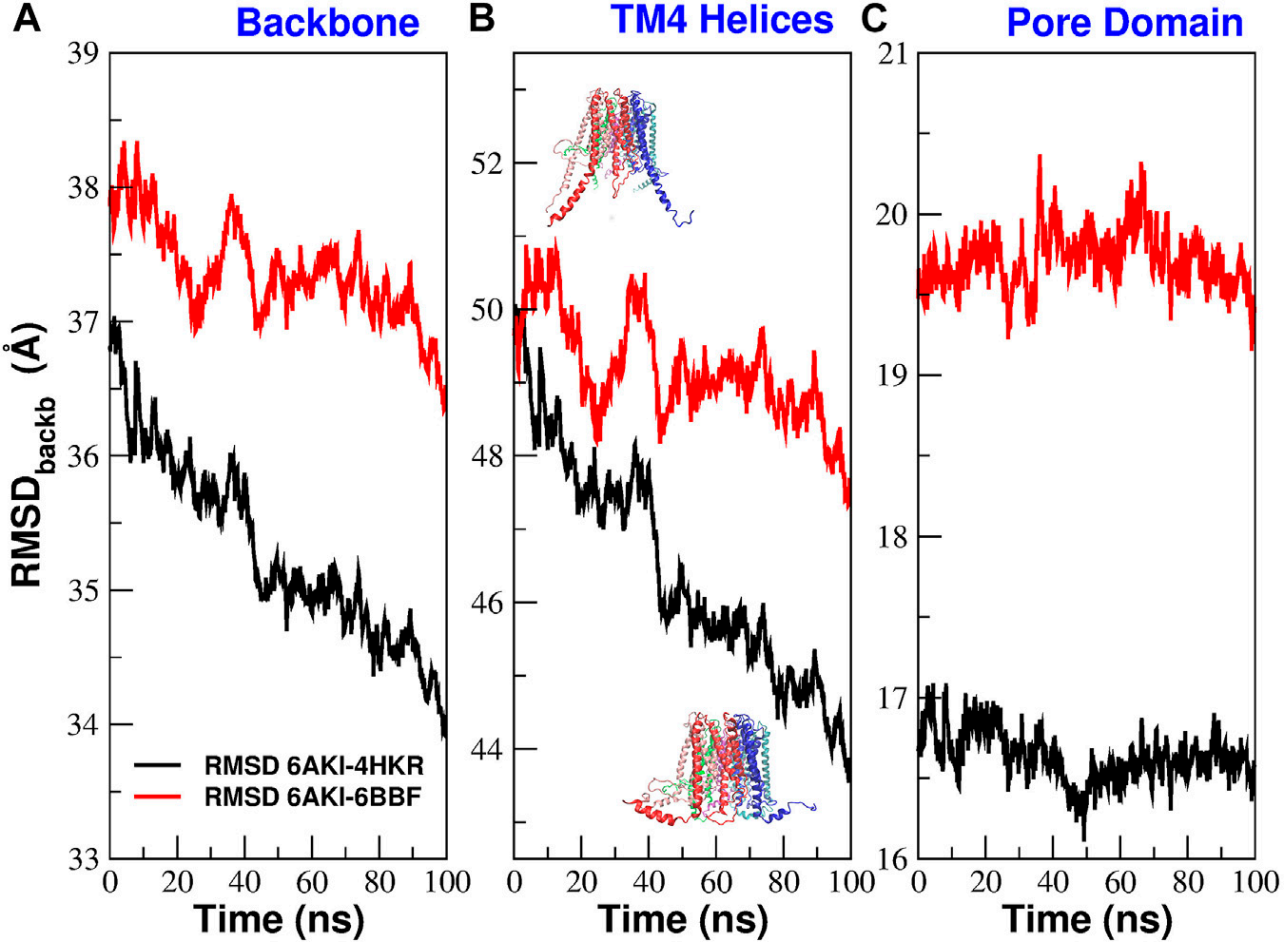
Evolution during the equilibrium simulation of the RMSD between 6AKI and the crystal structures of 4HKR and 6BBF. Structure superposition was performed using all backbone atoms. The actual RMSD was computed using different subsets of atoms. In panel **(A)** the RMSD was computed using all backbone atoms; in panel **(B)** it was computed using the backbone atoms of helices TM4; the overlayed structures represent the initial conformation of 6AKI with extended TM4 helices and the final configuration with bent TM4 helices; in panel **(C)** the RMSD calculation was performed using the backbone atoms of the pore helices.

Figure 5 reports the time evolution of the RMSD distance between 6AKI and the experimental structures of 4HKR and 6BBF during the equilibrium simulation of mutant P288L. In this calculation the two structures have been aligned using all backbone atoms while the actual RMSD calculation has been performed using all backbone atoms (Figure 5A), the backbone atoms of helices TM4 (Figure 5B) and the backbone atoms of the pore helices (Figure 5C). Figure 5A, shows that, during the equilibrium simulation, 6AKI becomes increasingly similar to both 4HKR and 6BBF but, unexpectedly for a putatively open structure, it is more similar to 4HKR than to 6BBF. Figure 5B shows that the increasing similarity between 6AKI and 4HKR is due to some structural rearrangement at the level of the outermost ring of helices. Indeed, as also shown in Supplementary Movie SM1, at the beginning of the simulation, TM4 helices of 6AKI are in an extended conformation, but during the run they spontaneously bend adopting a structural arrangement similar to that of the closed state. Finally, Figure 5C shows that no major structural rearrangement occurs at the level of the pore helices, even if, also in this district, 6AKI appears to be more similar to 4HKR than to 6BBF. The disruption of the bubble must thus be ascribed to some fine tuning of the pore conformation. In order to assess the variability across runs of the 6AKI-4HKR and 6AKI-6BBF RMSD, we performed a block average analysis (Frenkel and Smit, 2002) of the equilibrium simulation of 6AKI. Details can be found in the Supplementary Material section *Analysis of RMSD variability* where we also comment on the seemingly large RMSD values appearing in Figure 5.

The structural instability exhibited by 6AKI suggests that this structure is unlikely to represent the open state and more probably corresponds to an intermediate or a conformation on the slope of the free energy barrier which separates the closed state (4HKR) and the open state (6BBF). However, if we represent the free energy profile as a function of a single collective variable, a structural transition that brings 6AKI closer to 4HKR would automatically move it away from 6BBF and *vice versa* (Supplementary Figure S3A). This would be inconsistent with what observed in the RMSD analysis, i.e., that, during the simulations, 6AKI tends to get closer to both 6BBF and 4HKR. This seeming contradiction can be solved by considering that free energy is more realistically a function of several collective variables (Supplementary Figure S3B).

### 2.3 Targeted Molecular Dynamics Simulations

**FIGURE 6:**
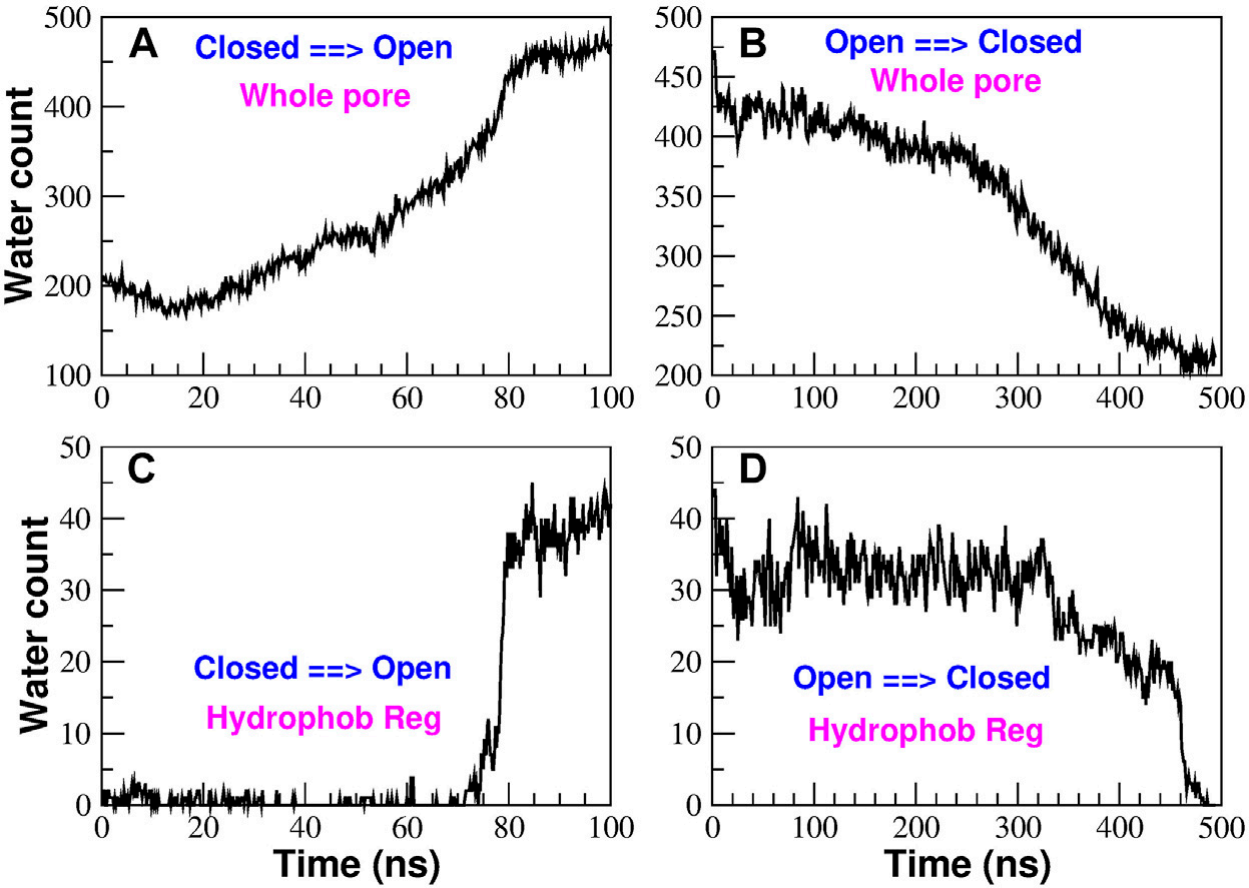
Time evolution of the water content in the pore region during the TMD simulations. Panels **(A)** and **(B)** show the number of water molecules in the whole pore region in the opening and closing simulation respectively. Panels **(C)** and **(D)** monitor the water count in the hydrophobic region on the opening [panel **(C)**] and closing [panel **(D)**] simulations.

In order to reproduce the conformational transition from the closed to the open state and *vice versa* we ran Targeted Molecular Dynamics simulations (Schlitter et al., 1994). Due to the structural instability of mutant P288L (PDB: 6AKI) (Liu et al., 2019) we chose as open state the structure of mutant H206A (PDB: 6BBF) (Hou et al., 2018), while the structure of the wild type solved by Hou et al (PDB: 4HKR) (Hou et al., 2012) was considered as representative of the closed state. Specifically, we ran two TMD simulations: a 100 ns simulation from the closed to the open state and a 500 ns simulation from the open to the closed state. As can be noted, these simulations are extremely long as compared to the typical length of TMD simulations reported in the literature (1 ns or less) (Schlitter et al., 1994; Xiao et al., 2015; Liang et al., 2017; Li et al., 2013). The length of our simulations was not required to induce the structural transition but rather for the destruction or formation of the bubble in the hydrophobic region of the pore. This behaviour is not completely unexpected considering that in TMD the biasing potential is applied to the protein structure while water molecules redistribute spontaneously as a result of the changing chemical environment around them. In addition, the formation/destruction of the bubble is typically a first order transition, which, once triggered by structural changes, can be irreversible on the simulation timescale (Giacomello and Roth, 2020); therefore very slow changes in the biasing potential are needed to avoid artifact. In this regard, it is also notable that the O → C simulation had to be 5 times longer than the C → O simulation. This suggests that the energy barrier in the O → C transition is higher than that in the C → O transition implying a lower free energy of the open state as compared to the closed configuration. The water content of the pore is monitored in Figure 6. If we consider the water count in the whole pore (Figures 6A,B), we can note that the number of water molecules increases or decreases in a rather gradual way. However, if we focus on the hydrophobic region only (Figures 6C,D), we observe an abrupt transition where the number of water molecules directly jumps from zero to the final value or vice versa. This behaviour suggests that bubble formation or destruction is a rare event possibly related to the overcoming of a high energy barrier. Moreover, this highly cooperative transition was also predicted in the thermodynamic model by Roth et al. (2008) where gating occurs in a narrow window of pore radii or hydrophobicities. Finally, the fact that bubble formation and destruction occurs at the end of the simulation both in the opening and closing transition, suggests the process to be strongly hysteretic, possibly due to the presence of hidden collective variables. This issue will be the object of a future investigation using techniques successfully applied in both model nanopores (Tinti et al., 2017; Camisasca et al., 2020) and biological ion channels (Costa et al., 2021). A more detailed description of the water and ion distribution in the pore is provided in the Supplementary Material section *Water and ion distribution during TMD simulations*.

### 2.3.1 Rotation of Pore Helices, SASA and Pore Radius

**FIGURE 7:**
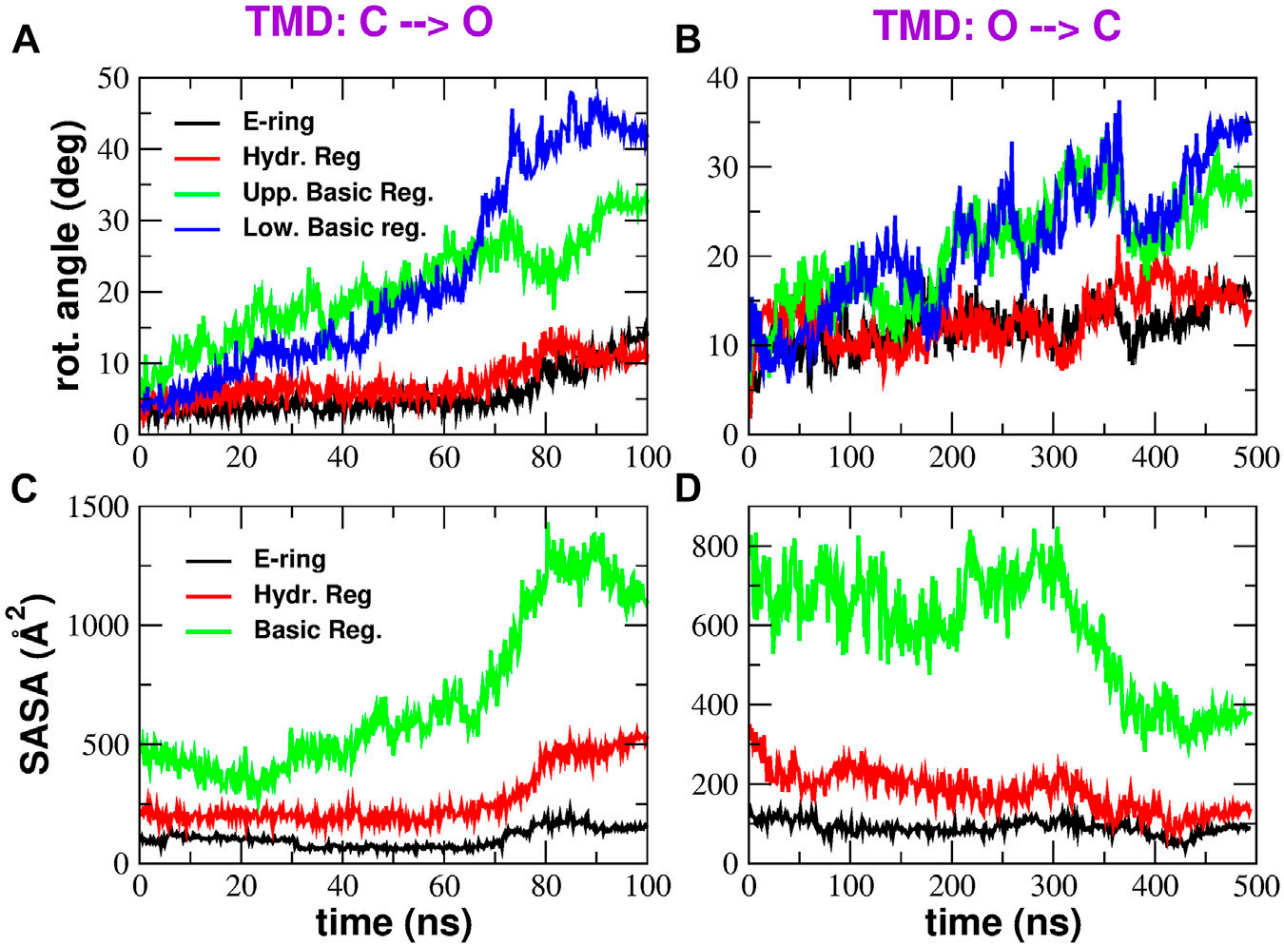
Rotation and Solvent Accessible Surface Area of pore helices. Panels **(A)** and **(B)** show the time evolution of the rotation angles (averaged over the six pore helices) on the opening and closing transition respectively. Rotation angles were measured at four different axial levels: E-ring (black curve), hydrophobic region (red curve), upper basic region (green curve) and lower basic region (blue curve). Panels **(C)** and **(D)** show the Solvent Accessible Surface Area in the opening and closing TMD simulation respectively. The black curve represents the SASA of the E-ring, the red curve is the SASA of the hydrophobic region and the green curve shows the SASA of the basic region.

A long-standing controversy in CRAC channel literature concerns the gating mechanism, that, according to some authors (Yamashita et al., 2017), is due to a rotation of pore helices; however, this event was not confirmed in a number of subsequent experimental (Hou et al., 2018; Liu et al., 2019) and computational works (Frischauf et al., 2019). Section *Rotation of pore helices* and Supplementary Figure S14 in the Supplementary Material show that the pore helices of CRAC channel have an intrinsic tendency to rotation that is present even during equilibrium simulations; in addition, the rotations seen in the experimental structures, reported in Supplementary Table S4, show good agreement with those observed in simulations. In the following we characterise rotation occurring during biased simulations. Figures 7A,B show that rotation of pore helices does occur during the TMD simulations. However, the rotation angles measured at different levels along the pore axis suggest that rotation does not occur as a rigid body rotation but rather as a twisting. It is also noteworthy that the rotation angles of the Glu-ring and hydrophobic region are much smaller than those of the basic region, presumably because the latter is located close to the N-terminus where chain constraints are expected to be weaker.

The value of the rotation angle of the hydrophobic region averaged along the last 20 ns of the C → O simulation is ∼11^*o*^ while the value averaged during the last 100 ns of the O → C simulation is ∼16^*o*^. These values are in reasonably good agreement with the average helix rotation of ∼15^*o*^ at residue F171 measured by Yamashita et al. (2017). It is important to note that, while our rotations were measured during a conformational transition from the open to the closed state and back, the rotation angles reported by Yamashita et al. (2017) are due to frequent spontaneous conformational fluctuations from the crystallographic structure that they measured during equilibrium simulations. This may be due to the fact that, even at equilibrium, the system transiently visits conformations relevant for its functional dynamics.

Finally, in the C → O transition it is possible to identify a change in slope of the rotation angles around 70 ns, just little before bubble destruction. This suggests that the rotation of pore helices might be the trigger for bubble destruction. However, the time coincidence between the change in rotation angles and bubble formation is less evident in the closing transition.

After the rotation of pore helices is ascertained, it is important to investigate the functional role of this event. According to the thermodynamic model by Roth et al. (2008), hydrophobic gating is under the control of two key parameters: the hydrophobicity of pore wall and the pore radius. We therefore checked whether either of these parameters was affected by pore helix rotation. Figures 7C,D show the time evolution of the Solvent Accessible Surface Area (SASA) in the most important regions of the pore: the Glu-ring, the hydrophobic region, and the basic region. It can be noted that, both in the opening and closing transition, the SASA plot shows a change in slope approximately at the same time of the change in slope of rotation angles. However, while the SASA change of the basic region and E-ring favour the increase of water content expected during opening and the decrease expected during closing, the SASA change of the hydrophobic region tends to oppose the wetting expected during opening and the de-wetting of the closing process. These results therefore suggest that the rotation of pore helices is not finalized to the creation of a thermodynamically favourable environment for bubble formation or destruction.

We now explore the possibility that the rotation of pore helices might cause a change of the pore radius. In Supplementary Figure S8, we consider two possible scenarios. In the first scenario, the rotation of pore helices is the main cause of the increase of the pore radius through a displacement of the bulky side chains of hydrophobic residues. In this case, if we compute a PMF as a function of rotation angle and pore radius, the low free energy region should be slanted. On the other hand, if we repeat the calculation after excluding the side chains from the calculation of pore radius, the low free energy region should be flat. In the second scenario, helix rotation and pore radius increase are simultaneous events but rotation is not the main cause of the growth of the pore radius. In this case, either performing the calculation with or without side chains, the low free energy region of the PMF should be sloped.

**FIGURE 8:**
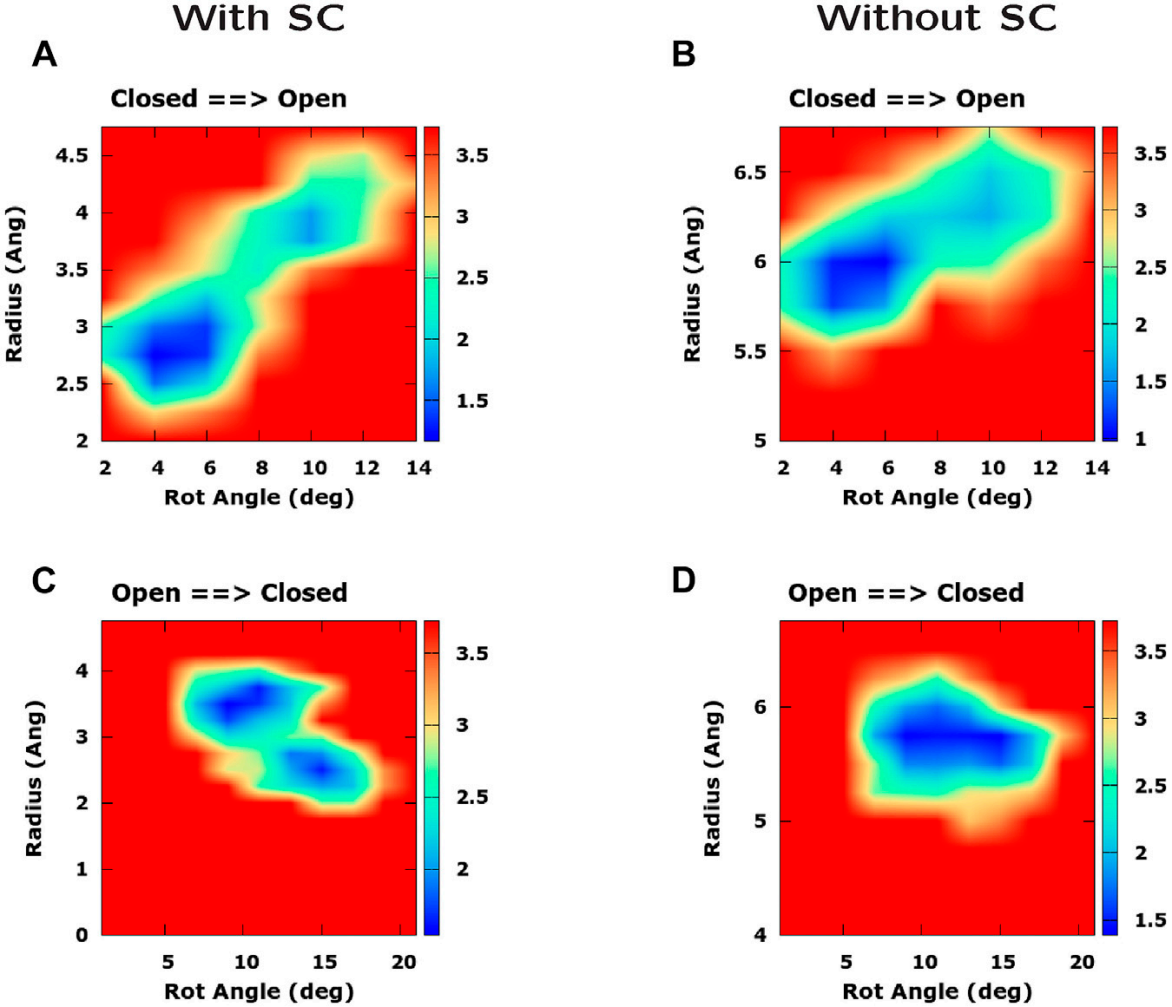
Influence of pore helix rotation on pore radius. The four panels represent a Potential of Mean Force as a function of pore radius and pore helix rotation angle. In panels **(A)** and **(C)** the PMF was computed including the side chain in the calculation of pore radius, whereas in panels **(B)** and **(D)** side chains were excluded from the calculation. Panels **(A)** and **(B)** refer to the opening TMD simulation while panels **(C)** and **(D)** refer to the closing simulation. Free energies are expressed in kcal/mol.

This strategy can be employed to analyze the PMFs derived from our TMD simulations and illustrated in Figure 8. In the opening simulations, both computing the PMF with (Figure 8A) and without side chains (Figure 8B), the minimal free energy region is inclined. This suggests that helix rotation and radius enlargement occur simultaneously but there is no causal link between the two phenomena. However, if we consider the closing simulation, we note that, when the PMF is computed using side chains (Figure 8C), the increase of the rotation angle is accompanied by a decrease in the pore radius while this effect is suppressed when removing the side chains (Figure 8D). The conclusion of these results is that rotation of pore helices might play some role in the change of pore radius. However, this driving force is probably not the only one and the change of pore radius also depends on helix displacement.

### 2.3.2 Contact Analysis

**FIGURE 9:**
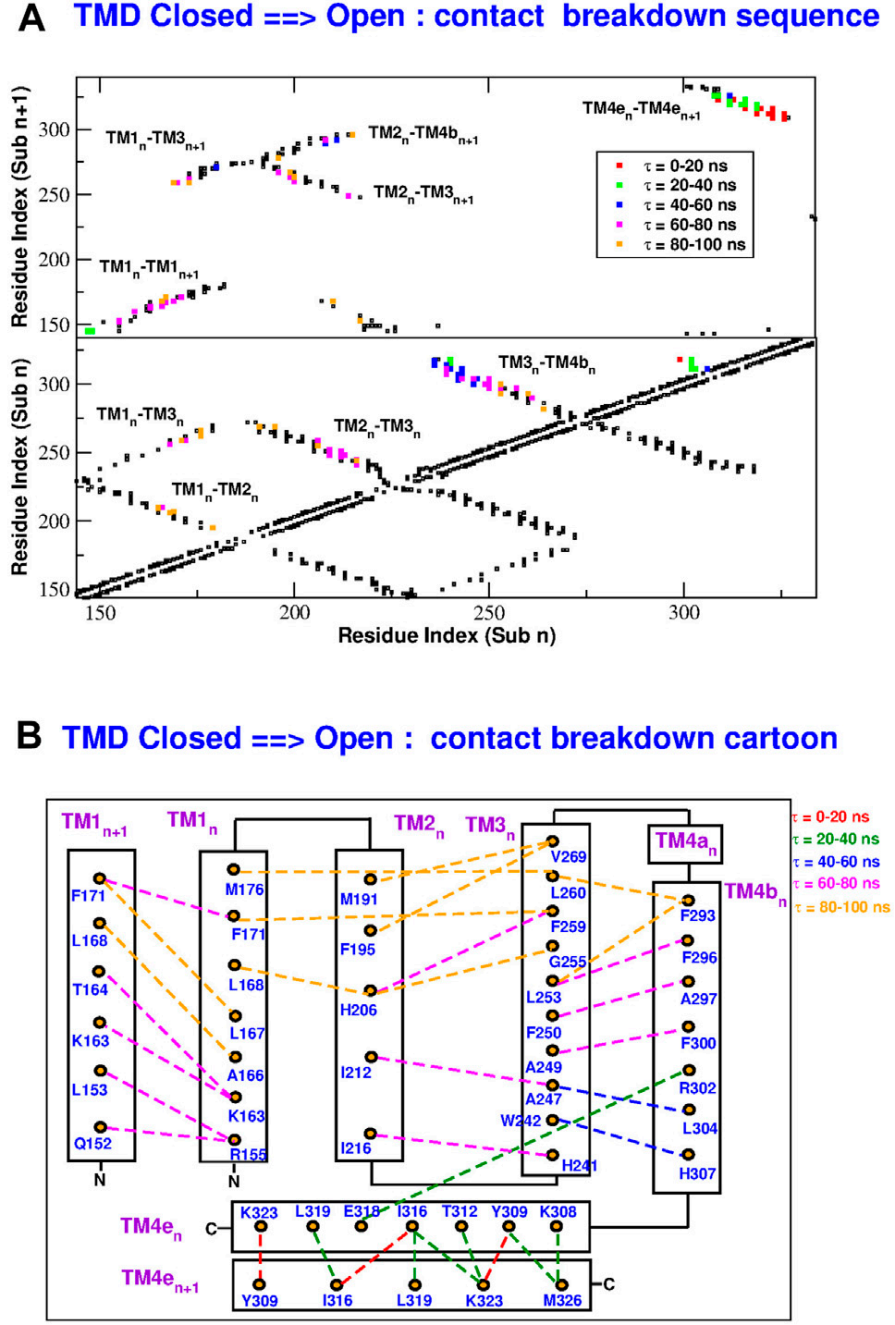
Sequence of contact breakdown in the TMD opening simulation. Panel **(A)**: contact map. Black squares are contact present in the closed state and not broken during the simulation. Coloured squares are contacts broken at different times *τ* during the simulation. The lower part of the map shows intra-subunit contacts, the upper part contacts between subunits *n* and *n* + 1. Panel **(B)**: cartoon representation of contact breakdown. Broken contacts are represented by dashed lines with color-coded breakdown time *τ*.

**FIGURE 10:**
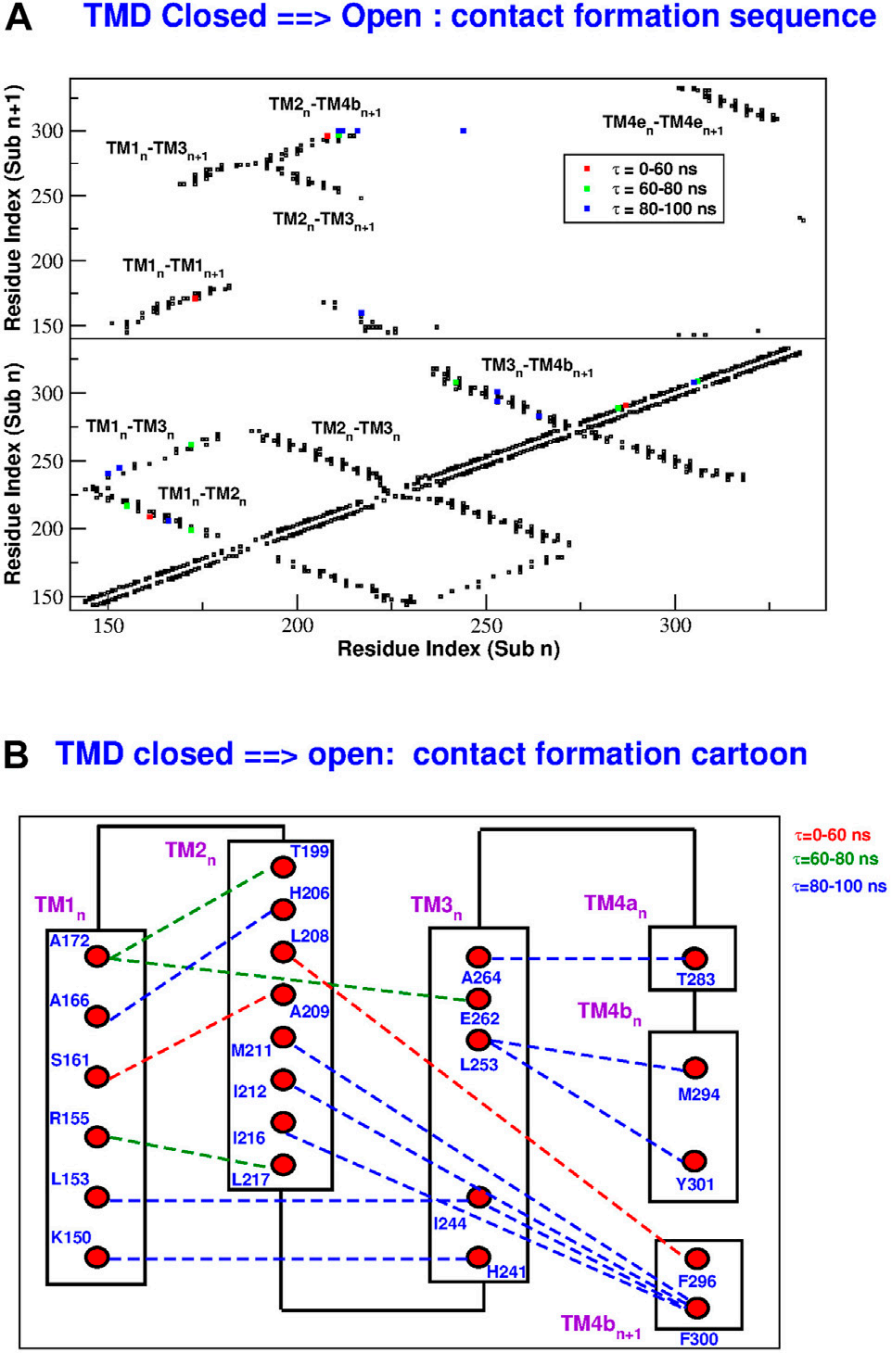
Sequence of contact formation in the TMD opening simulation. Panel **(A)**: contact map. Black squares are contact present in the closed state and not broken during the simulation. Coloured squares are contacts formed at different times *τ* during the simulation. The lower part of the map shows intra-subunit contacts, the upper part contacts between subunits *n* and *n* + 1. Panel **(B)**: cartoon representation of contact formation. Formed contacts are represented by dashed lines with color-coded formation time *τ*.

In order to reconstruct the gating mechanism, for each frame of the TMD trajectories we built a contact map (two residues are considered to be in contact if at least two heavy atoms of the side chains are closer than 5.0 Å). In this way it is possible to determine the sequence of contact breakdown (Figure 9 and Supplementary Figure S9) and formation (Figure 10 and Supplementary Figure S10). The black squares in the maps represent contacts present in the initial conformation and not broken during the simulation. The coloured symbols represent contacts formed or broken at different times during the simulation. In particular, we only considered contacts formed or broken in at least 3 of the subunits and we averaged their formation or breakdown times. In the contact maps, the elements on the diagonal represent contacts inside the same alpha helix. The elements orthogonal to the diagonal represent contacts between different alpha helices of the same subunit. Finally the islands of off-diagonal elements represent inter-subunit contacts. The maps show that all the broken and formed contacts are either intra-subunit or between neighbouring subunits *n* and *n* + 1.

During the opening simulation, the number of broken contacts is much higher than the number of formed contacts, consistent with the formation of a looser structure. As shown in Figure 9, the contact breakdown originates at the coiled coil between TM4-ext helices of neighbouring subunits and propagates towards the extracellular side through the TM3-TM4b interface. The contact breakdown then propagates towards the inner rings of helices. In particular we can observe the breakdown of contacts in the upper part of the TM2-TM3 and TM1-TM2 interface and a breakdown of contacts spanning the whole length of neighbouring TM1 helices. The few formed contacts (Figure 10A) are scattered throughout the contact map, but indeed they are very localized in space (Figure 10B). Most of them, indeed, involve the lower part of helices TM2(n)-TM4b(n + 1) and TM1(n)-TM3(n). This opening mechanism is consistent with the fact that activator STIM1 is known [Hou et al. (2012) and references therein] to break down the TM4e(n)-TM4e(n + 1) coiled-coil to form a new one. The conformational change is then expected to allosterically propagate (Yeung et al., 2018) to the innermost circle of helices where gating occurs.

The closing process, depicted in Supplementary Figure S9 and Supplementary Figure S10, is basically the reverse of the opening one. In this case the number of formed contacs is much higher than that of the broken ones. As illustrated in Supplementary Figure S10, contact formation starts in the extracellular side of helices TM3-TM4b and propagates downwards to restore the coiled coil between neighbouring TM4e helices. Contact formation also propagates towards inner helices. In both the opening and closing processes, the last events involve H206 on helix TM2. As shown in Figure 9B, during opening H206 breaks its hydrogen bond with L202 (TM2) and breaks the hydrophobic contacts with L168 (TM1) to form new hydrophobic contacts with A166 (TM1). Other broken contacts are those with F259 and G255 of TM3 (Figure 10B). The fact that the contacts involving H206 are formed and broken at the very end of the process, suggests that the whole mechanism is finalized to these events highlighting the importance of H206. This is consistent with the experimental evidence (Yeung et al., 2018) that most mutations of H206 create constitutively open mutants. Indeed our open structure was taken from the backmutated H206A mutant (Hou et al., 2018).

### 2.3.3 Analysis of Inter-helical Distances

The contact analysis has highlighted two main events during the gating process: the massive formation or breakdown of contacts between helices TM3 and TM4b and the final formation and breakdown of several contacts involving H206. We will now analyze more in depth these two events starting with the formation/destruction of contacts in the TM3/TM4b interface.

**FIGURE 11:**
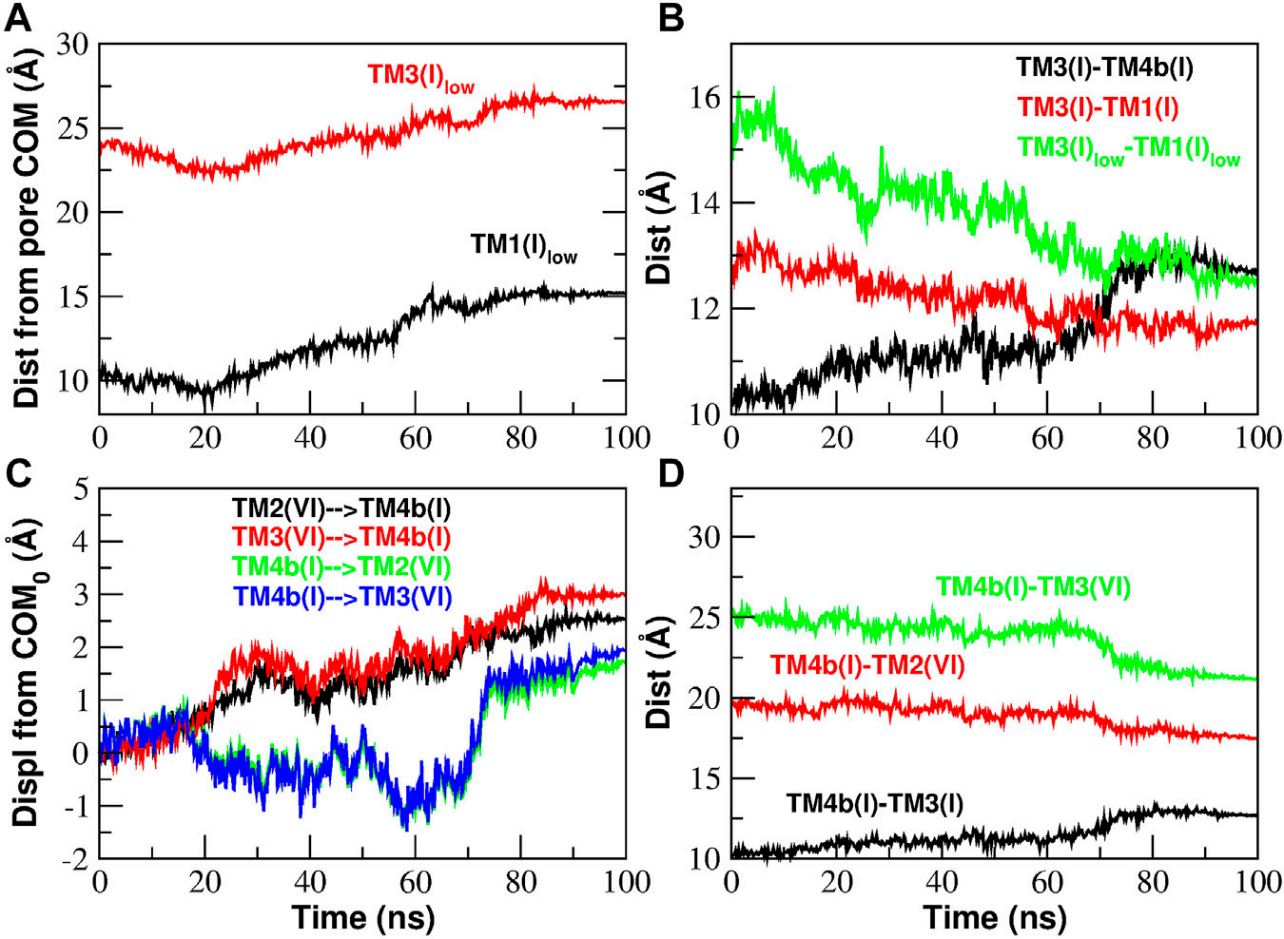
Inter-helical distances involving helices TM3 and TM4b of Subunit I during the opening TMD simulation. Panel **(A)**: distance of the lower part of helices TM1 and TM3 from the Center of Mass (COM) of the pore. Panel **(B)**: the increase of the distance TM3-TM4b is paralleled by a decrease of the TM3-TM1 distance (more pronounced if considering only the lower part of the two helices). Panel **(C)**: displacement of the center of mass of helices TM4b(I), TM2(VI), TM3(VI) from its initial position. The displacement vector is projected such that a positive displacement represents an approach while a negative displacement a distancing between the helices. Panel **(D)**: the increase of the distance TM3(I)-TM4b(I) is paralleled by a decrease of the TM4b(I)-TM3(VI) and TM4b(I)-TM2(VI) distances.

In the C → O transition, after the massive breakdown of contacts, helices TM3 and TM4b move apart (Figure 11B) and they face a different destiny. Helix TM3, in particular its lower part, moves centrifugally. The displacement of helix TM3 makes room for a corresponding centrifugal displacement of the lower part of helix TM1: this is the opening of the basic region of the pore. These motions are described in Figure 11A where we show the displacement of helices TM1 and TM3 from the center of mass of the pore. Both helices move centrifugally, but since TM1 takes a longer step, the TM1-TM3 distance decreases (Figure 11B) justifying the formation of contacts that we observed in the contact analysis (Figure 10).

We now turn to the fate of TM4b. In Figure 11C we show the displacement of TM4b(n + 1), TM3(n), and TM2(n) with respect to the initial position of the centers of mass. The displacements have been projected on the vectors connecting the COMs of the three helices so that a positive displacement corresponds to an approach while negative displacements correspond to a distancing. Our plots show that, while TM4b(n + 1) moves closer to TM2(n) and TM3(n), also TM2(n) and TM3(n) move towards TM4b(n + 1) so that there is a decrease of distances TM4b(n + 1)-TM3(n) and TM4b(n + 1)-TM2(n) as illustrated in Figure 11D. This pattern is in agreement with the TM4b(n + 1)-TM2(n) contact formation observed in the contact analysis (Figure 10).

As shown in Supplementary Figure S11, what happens during the closing transition is the reverse of what occurs during opening. The lower parts of helices TM1 and TM3 move towards the center of the pore (Supplementary Figure S11A) and since TM1 takes a longer step, the distance between the two helices increases (Supplementary Figure S11B) and we have breakdown of TM1-TM3 contacts (Supplementary Figure S9). We also note that, as helices TM4b(n + 1), TM2(n) and TM3(n) move apart from each other (Supplementary Figure S11C), their distance increases (Supplementary Figure S11D) and we have the breakdown of TM4b(n + 1)-TM2(n) contacts highlighted in the contact analysis (Supplementary Figure S9).

**FIGURE 12:**
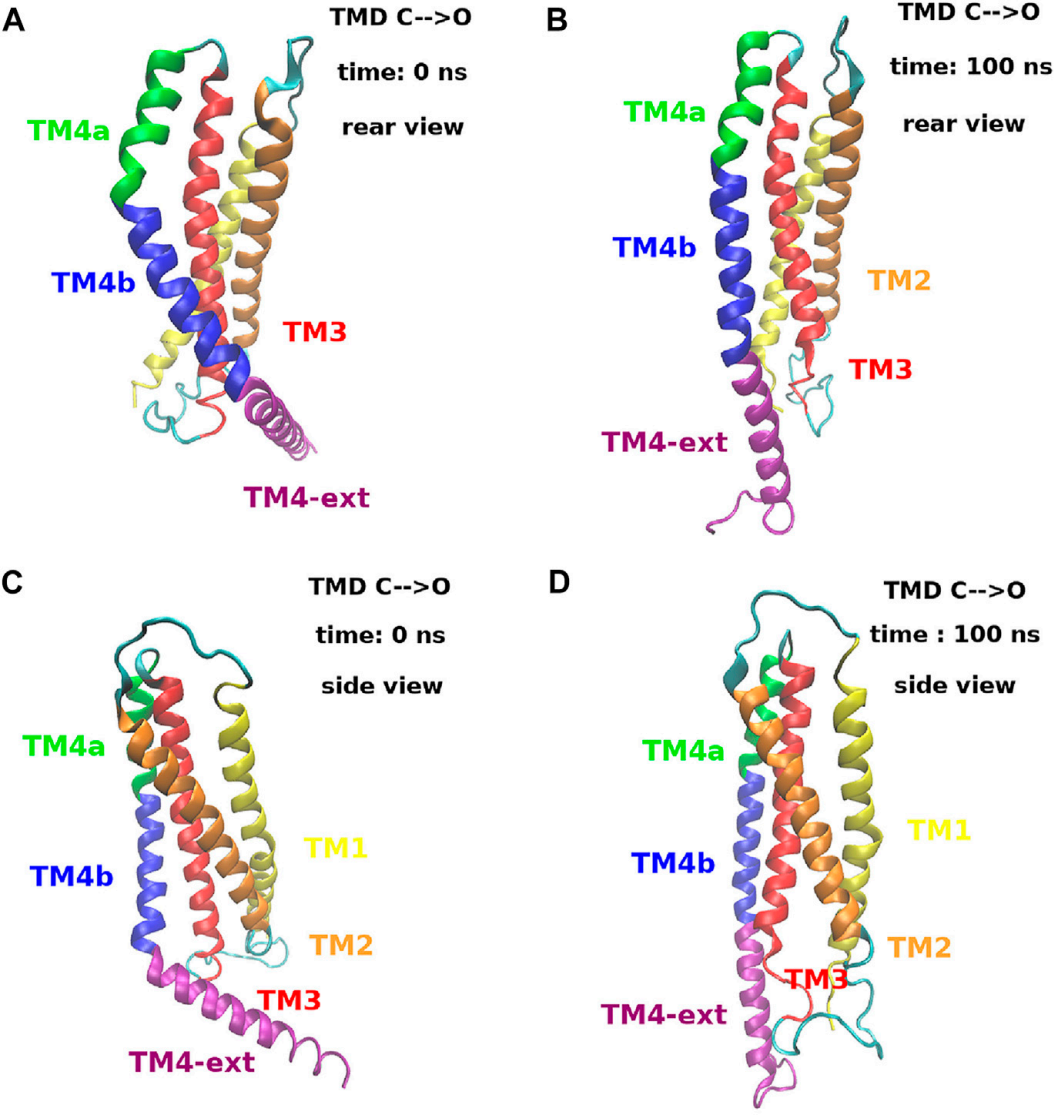
Extension process of helix TM4 during the opening TMD simulation. Panels **(A)** and **(B)** show a rear view of a single subunit at time *t* = 0 ns and *t* = 100 ns. Panels **(C)** and **(D)** show a side view of the same subunit at the same simulation times. The color code is the following. Helix TM1: yellow, helix TM2: orange, helix TM3: red, helix TM4a: green, helix TM4b: blue and helix TM4-ext: purple. A dynamic view of this process can be seen in Supplementary Movie S2.

The structural mechanism that underlies the distance plots in Figure 11 is depicted in Figure 12. Panels (A) and (B) of Figure 12 show a rear view of a single subunit at time zero and at 100 ns during the opening process. At time zero, in the closed state, helix TM4 has a double bend, one between TM4a and TM4b and one between TM4b and TM4-ext. In this particular arrangement it can be observed that helices TM4b and TM4-ext are behind helix TM3 that therefore is prevented from moving back. At time 100 ns, helix TM4 is fully extended. In this conformation, the space behind helix TM3 has been cleared and, since helix TM4b is no longer in the way, helix TM3 can move backwards. Panels (C) and (D) of Figure 12 show a side view of the same process. What is notable here is that the extension of TM4b allows the backward movement of the lower part of TM3. This motion, in turn, clears the space behind TM1 allowing a backward movement of the lower part of this helix that opens the basic region of the pore. This process is dynamically illustrated in Supplementary Movie S2.

**FIGURE 13:**
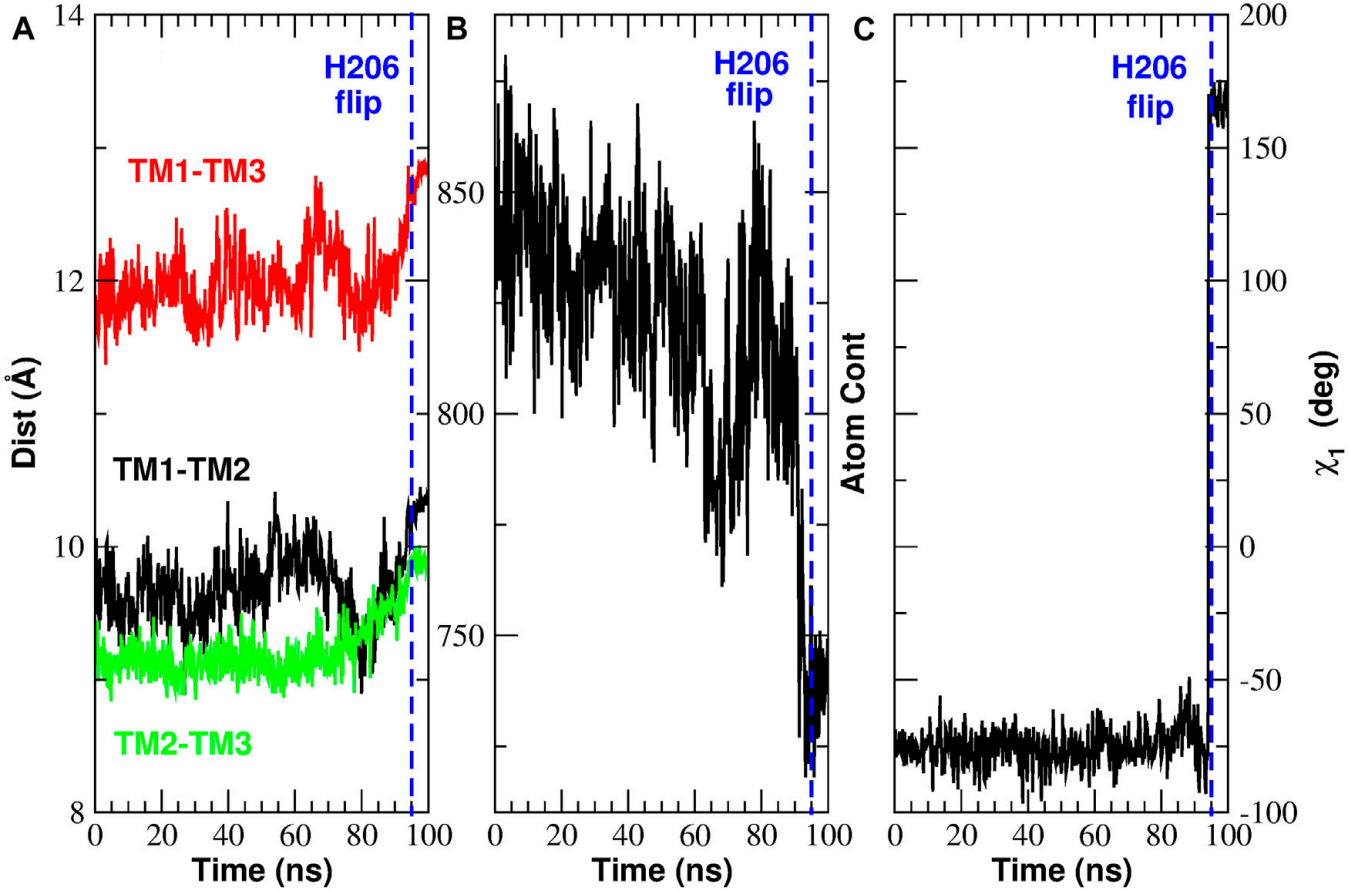
Rearrangement of H206 during the opening TMD simulation. Panel **(A)**: inter-helical distances TM1-TM2 (black curve), TM1-TM3 (red curve) and TM2-TM3 (green curve). Panel **(B)**: number of atomic contacts of H206 as defined by a distance cutoff of 7.5 Å. Panel **(C)**: dihedral angle *χ*_1_ of H206.

As already discussed, the contact analysis has highlighted two key events. After analyzing the implications of the TM3-TM4b contact breakdown, we now turn to the events involving H206. In Figure 11 and in Supplementary Figure S11 we have observed a significant rearrangement of helices TM1, TM2, TM3. In particular, we have noted that, during the opening process, helix TM1 moves centrifugally while helices TM2 and TM3 of subunit *n* move towards helix TM4b of subunit *n* + 1. Figure 13A shows that these events cause an increase in the distances TM1-TM2, TM1-TM3, and TM2-TM3. Since the side-chain of H206 leans in the space between these three helices, their distancing causes a decrease in the atom density around H206, see Figure 13B showing the number of atom contacts of H206 as defined by a distance cutoff of 7.5 Å. Finally, Figure 13C shows that as soon as the atom density around H206 decreases, the side chain of this residue swings due to a rotation around dihedral angle *χ*_1_. Supplementary Figure S12 shows that the reverse process occurs during closing. In this case there is a decrease of the distances, TM1-TM2, TM1-TM3, and TM2-TM3 (Supplementary Figure S12A). This causes an increase in the atom density around H206 (Supplementary Figure S12B) so that the side chain of this residue is pushed back (Supplementary Figure S12C) to the conformation typical of the closed state.

**FIGURE 14:**
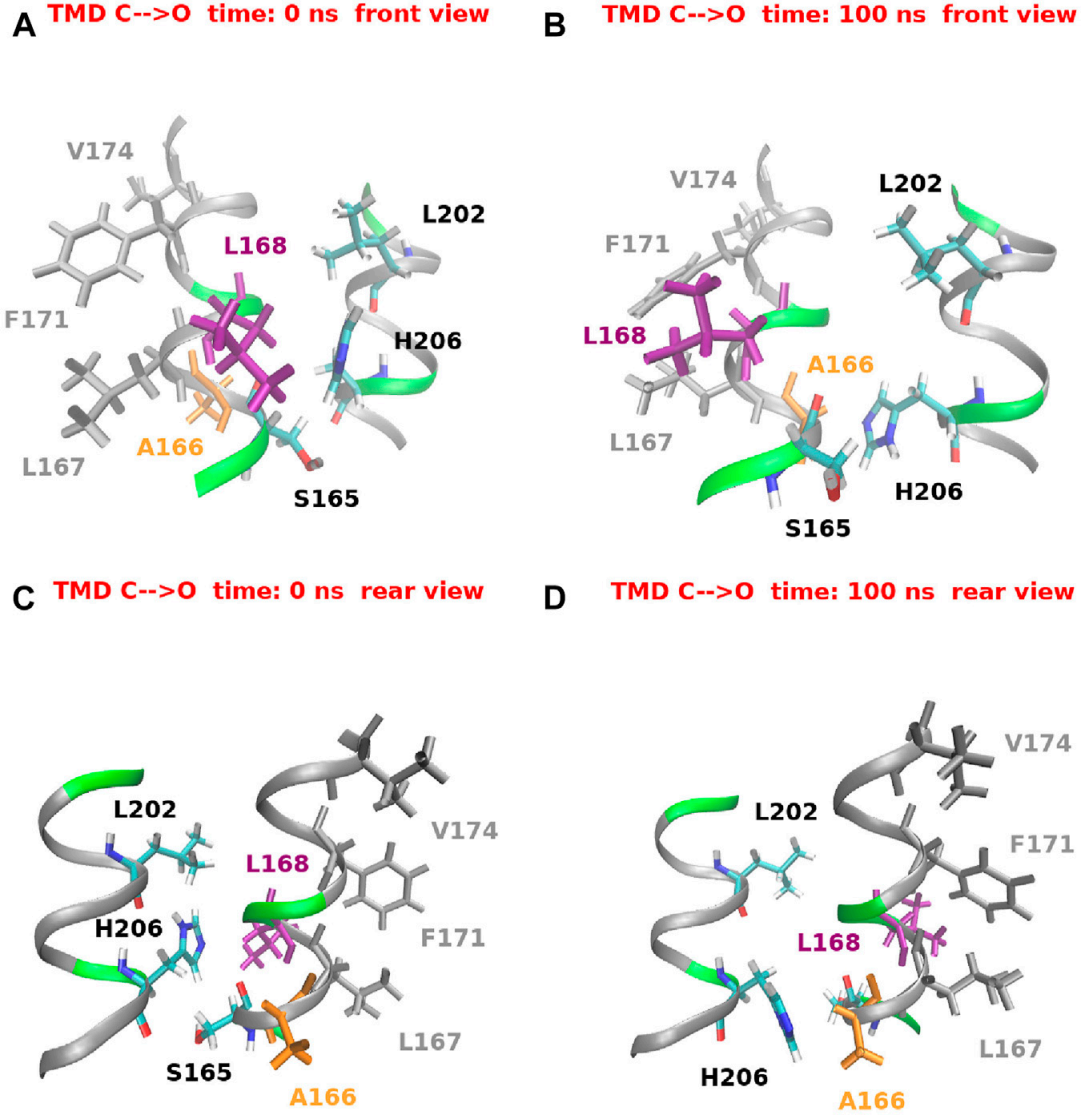
Histidine 206 swing during the opening TMD simulation. For the sake of clarity only a small stretch of helices TM1 (residues 165–175) and TM2 (residues 200–208) is shown. Panels **(A)** and **(B)** show a front view at simulation time *t* = 0 ns and *t* = 100 ns respectively. Panels **(C)** and **(D)** show a rear view of the same helices at the same simulation times.

Figure 14 provides a structural view of the conformational transition involving H206. For the sake of clarity only a short stretch of helices TM1 and TM2 is shown. Figures 14A,B offer a view from the front while Figures 14C,D from the rear. In the closed state, as predicted by Yeung et al. (2018), H206 is hydrogen bonded with L202 also located on TM2. H206 is also close to S165, but differently from Frischauf et al. (2019), no hydrogen bond can be seen. Finally, in the closed state, H206 forms hydrophobic contacts with L168 on helix TM1. When opening occurs, H206 rotates around the *χ*_1_ dihedral breaking the hydrogen bond with L202 and swinging from the contact with L168 to a hydrophobic contact with A166 (also located on TM1). It is important to note that this rearrangement of H206 creates a free space behind the hydrophobic region of TM1. A dynamic view of this process is provided by Supplementary Movie S3.

## 3 Discussion

In this work we computationally characterized the closed and two putatively open conformations of CRAC channel to shed light on its gating mechanism. The assignment to the open or closed state of the available structures is not completely obvious. Indeed, the radius profile of structure 4HKR is always larger than the radius of a single water molecule. This means that, from a geometric standpoint, the pore is water-accessible and it should be assigned to the open state in disagreement with the results of the fluorescence-based flux measurements performed on the purified channel reconstituted in liposomes (Hou et al., 2012). This contradiction can be reconciled if the geometric analysis is integrated with a study of the chemical properties of the pore wall. Our equilibrium simulations show a peak of the hydrophobicity profile in correspondence of the hydrophobic region, where also the Potential of Mean Force of water has its maximum. This suggests a scenario of functional occlusion of the pore even in the absence of steric block, which is the hallmark of *hydrophobic gating* (Aryal et al., 2015). During our equilibrium simulations of the closed state 4HKR, a vacuum bubble appears early in the hydrophobic region and is stably maintained throughout the simulation. The bubble prevents the flow of liquid water and ions that would be obliged to release their hydration shells. In agreement with this pattern, during our TMD simulation the bubble forms in the O → C transition and disapperars in the C → O transition. According to the thermodynamic theory by Roth et al. (2008), even in a geometry as complex as that of KcsA channel, hydrophobic gating is tuned by pore radius and wall hydrophobicity. The phenomenon is predicted to be highly cooperative and to occur abruptly in a narrow range of values of the radius or hydrophobicity (in agreement with our Figures 6C,D). Roth et al. (2008) arrive as far as to say that all ion channels use hydrophobic gating, the only difference regarding the details of the conformational change adopted to tune radius and hydrophobicity of the pore. This statement probably over-estimates the diffusion of hydrophobic gating. However, hydrophobic gating, originally discovered in simple model nanopores (Beckstein and Sansom, 2003), has recently been identified in an increasing number of biological ion channels (see Aryal et al. (2015) and references therein) including bacterial mechanosensitive channels, ligand-gated ion channels of the Cys-loop family (including the nicotinic acetylcholine receptor, GLIC, and 5-HT_3_ receptor), and even members of the superfamily of tetrameric P-loop cation channels.

The functional assignment of the P288L mutant (PDB: 6AKI) is just as controversial. The dOrai P288L mutant is the equivalent of the P245L gain of function mutant (Nesin et al., 2014) of hOrai1 that causes tubular myopathy and congenital miosis. Moreover, when Liu et al. (2019) performed patch clamp measurements on HEK cells transfected with the P288L mutant, they recorded an inward-rectified Ca^2+^ current that in the absence of calcium was replaced by a current of monovalent cations. However, Hou et al. (2020) were unable to detect ion permeation through purified P288L Orai in liposomes. Hou et al. (2020) also observed that the narrow pore radius profile of P288L made this structure similar to the non-conductive *unlatched-close* conformation discovered by their group. Another piece of evidence is represented by the scanning mutagenesis study by Yeung et al. (2018). This study revealed that P245C, along with many other mutants of Orai1, displays a basal level of constitutive activation that can be enhanced by STIM1 interaction. Indeed, the STIM1-induced increase of current shows an inverse relationship with constitutive activity. These results suggest that open mutants are not maximally active but rather adopt one or more states that can be further activated by STIM1. This scenario is consistent with our equilibrium simulations where 6AKI structure is conformationally unstable undergoing a spontaneous bending of TM4 helices and the disruption of a bubble initially present in the hydrophobic region. We thus suggest this structure represents an intermediate in the gating process rather than the open state conformation. The P288L structure resolved by Liu et al. (2019) is thus likely to represent one of the metastable states postulated by Yeung et al. (2018) whose activity can be enhanced by STIM1.

CRAC gating mechanism is a highly controversial issue. In a seminal work Yamashita et al. (2017), on the grounds of Cd^2+^ accessibility experiments on cysteine mutants of pore wall residues, proposed a mechanism based on a modest counter-clockwise rotation of pore helices aimed at displacing from the pore lumen the bulky side-chains of hydrophobic residues. This model was also supported by equilibrium MD simulations (Yamashita et al., 2017) of wild type and mutant channels revealing frequent spontaneous conformational fluctuations involving rotation of the pore helix relative to the crystallographic structure. The rotation of F99 was also observed in MD simulations performed by Bulla et al. (2019) on the H134C constitutively open mutant. The rotation of pore helices, however, was not observed in a number of subsequent computational and experimental works. Frischauf et al. (2019) in a molecular dynamics simulation study of a homology model of human CRAC channel did not observe any rotation of pore helices. Neither was any rotation observed in the crystal structure of the putatively open mutant P288L (Liu et al., 2019). Recently the crystal structure of another constitutively open mutant, H206A, has been resolved (Hou et al., 2018). The resolution (6.7 Å) however, was so low that side chains were not visible making it impossible to ascertain a possible rotation. When the structure was resolved again (Hou et al., 2020) at 3.3 Å resolution through cryo-electron microscopy, rotation was not observed and the data suggested a simple dilation of pore helices. However, it must be noted that, in order to increase the resolution of the cryo-EM structure, the mass of CRAC channel was increased through formation of a complex with the Fab fragment of a monoclonal antibody. The binding site of the antibody is the extra-cellular loop connecting helices TM2 and TM3 and the close proximity of the docking point with the pore helices might prevent their rotation. Even if Hou et al. (2020) showed that acute application of the antibody does not alter Ca^2+^ influx, the short incubation time of the antibody-channel mixture may have led to a strained conformation limiting the mobility of pore helices.

Our Targeted Molecular Dynamics simulations showed a rotation of pore helices occurring as a twisting, rather than a rigid body rotation. Interestingly, the rotation angle plot exhibited a change in slope matching the time of formation or disruption of the bubble in the hydrophobic region. The rotation of the hydrophobic region however, induced a change of the Solvent Accessible Area that opposed rather than favouring the formation of the bubble. Further analysis revealed that helix rotation might play a role in the change of pore radius even if helix displacement is also important. In other words our work suggests that the purpose of pore helix rotation is not to create a thermodynamically favourable environment for bubble formation or disruption but it contributes to change the pore radius complementing the effect of helix displacement.

A fascinating feature of CRAC channel is the recently proposed (Zhou et al., 2019; Yeung et al., 2020) allosteric nature of its activation mechanism. The activator protein STIM1 is known to bind to the TM4-ext helices, the outermost ring of helices. This event then induces opening of the pore that is lined by helices TM1, the innermost ring of helices. The coupling between these two events occurring far away in space implies the propagation of a conformational wave.

The allosteric nature of CRAC gating is also suggested by a recent work of Bulla et al. (2019) on a homology model of human CRAC channel. In particular, it was shown that the tubular myopathy related mutation T184M, on helix TM3, favours channel opening but is strictly dependent on STIM1. The mutation thus, does not lock the pore in a permanently open state but favours the propagation of the conformational change triggered by STIM1 docking. This finding is consistent with our results. Indeed, residue T184 in human CRAC corresponds to residue L256 in the *Drosophila* variant and our contact analysis revealed that the neighbouring residue G255 is involved in the breakdown or formation of contacts with the critical H206 residue in the final stage of the process. In order to shed light on this mechanism, we systematically applied this approach and we thus determined the sequence of contact breakdown and formation during the C → O and O → C transitions. We discovered that in the opening process contact breakdown starts at the coiled-coil between TM4-ext helices (where STIM1 is expected to bind) and proceeds to the extracellular side across the TM3-TM4b interface. The breakdown wave then further proceeds to the inner helices. What is more interesting, both in the opening and closing processes, the final stage involves formation and breakdown of contacts of H206 suggesting that the whole mechanism might be finalized to this event. The importance of H206 is well documented in the literature (Yeung et al., 2018). Its mutations to smaller and/or hydrophylic residues (like Ala, Cys, Ser, Thr) generate Ca^2+^ selective, constitutively open mutants.

The analysis of our TMD simulations reveals that, in the opening process, the extension of helix TM4 causes a massive breakdown of contacts in the TM3-TM4b interface that leads to the separation of the two helices. In particular, helix TM4b moves aside clearing the space behind TM3 that can move back. This event, in turn, clears the space behind the lower part of helix TM1 that also moves back, increasing the radius of the basic region and drawing water from the bulk. In this last stage, a number of TM1-TM3 contacts are established. The transduction pathway TM4b → TM3 → TM1 (basic region) was also predicted by Liu et al. (2019) based on the analysis of the structure of mutant P288L. The existence of this pathway was validated in patch-clamp and Ca^2+^ flow measurements of a series of mutants (Liu et al., 2019). For instance, the mutation of residues L153, S154, and K157 that are involved in the TM1-TM3 interactions, significantly reduced the extracellular Ca^2+^ influx.

The rearrangement of helices TM1, TM2, and TM3 determines in our opening TMD simulations an increase of the inter-helical distances TM1-TM2, TM1-TM3, and TM2-TM3. This results in a decrease of the atomic density around H206 whose side chain leans into the space delimited by the three helices. As soon as the atomic density around H206 is sufficiently low the side chain of H206 swings from a position in hydrophobic contact with L168 to a new position where it forms a hydrophobic contact with A166. It is important to note that the rearrangement of H206 creates a free space behind the hydrophobic region of helix TM1.

The functional role of H206 is object of debate in the literature. According to Frischauf et al. (2019) H206 plays its role by hydrogen bonding S165 and S169 on helix TM1 so as to stabilize the closed state. These bonds, however, can only be found in less than 5% of the frames of our simulations. In an alternative model proposed by Yeung et al. (2018), H206 is hydrogen bonded with the backbone carbonyl of L202, in agreement with our TMD simulations. Moreover, in a series of mutagenesis experiments, Yeung et al. (2018) showed that the ability of the residue in position 206 to stabilize the closed state does not depend on its hydrogen-bonding ability but rather on the side chain volume. In other words H206 would act as a *steric brake* pushing against helix TM1 and preventing its backward movement. In agreement with this model we speculate that the void space created by the flipping of H206 observed in our TMD simulations would allow the backward motion of the hydrophobic region of TM1. According to the themodynamic theory by Roth et al. (2008) even a small increase in the pore radius could then be sufficient to break the bubble and open the pore.

Even if in our O → C simulation the displacement of H206 takes place just before the formation of the bubble, in the C → O simulation the H206 swing occurs at time *t* ∼95 ns, after the bubble has already been destroyed. Moreover in neither simulation do we observe any change in the radius of the hydrophobic region after the H206 swing. This pattern however, may be due to an insufficiently long Targeted MD simulation or to the limitations of the TMD algorithm. A more accurate (albeit more computationally expensive) enhanced sampling approach like Transition Path Sampling (Bolhuis et al., 2002) might have reproduced a behaviour consistent with the steric brake model.

The mechanism of conformational transition from the closed to the open state emerging from our simulations, sheds some light on the elusive role of the *unlatched-closed* conformations. These structures (PDB: 6BBG and 6BBH), featuring extended TM4 helices and a pore domain (TM1-TM4a) indistinguishable from that of the closed state (PDB: 4HKR) within the limits of the diffraction data (Hou et al., 2018), suggest that the unlatching of TM4 helices does not automatically lead to pore opening. Indeed, Hou et al. (2018) suggested that unlatching is a necessary but not sufficient condition for pore opening. This result significantly complicates the formulation of a convincing transition mechanism, but the problem may be solved by adopting a dynamic rather than a static view. Hou et al. (2018) put forward the idea that the unlatched-closed conformations may represent intermediates in the path from the closed to the open state. These authors suggested that, even in the closed state, a chemical equilibrium should exist between the bent and extended conformations of helix TM4. The interaction with the STIM1 activator would then shift the equilibrium to the extended state generating an unlatched-open conformation. The propagation of the conformational change to the inner rings of helices would then complete the transition to the closed state. The results of our equilibrium simulation of 6AKI perfectly fit within this scenario. As already discussed, 6AKI features extended TM4 helices and a pore radius profile similar to that of the closed state. The structure of 6AKI is therefore very similar to that of the unlatched-closed conformations resolved by Hou et al. (2018). The spontaneous bending of TM4 helices observed in our equilibrium simulations highlights the conformational instability of 6AKI supporting the idea that this structure, as conjectured for unlatched-closed conformations, is an intermediate, presumably very close to the quiescent state along the gating pathway. Our TMD simulations, on the other hand, highlight a sequence of events that causally link the unlatching of helix TM4 with the dissolution of the bubble *via* the backward motion of helices TM3 and TM1, the distancing of helices TM1, TM2, and TM3, the swinging of the side-chain of H206 and the increase of pore radius in the hydrophobic region. In short sum, our work confirms with dynamical data of MD simulations the sequence of channel activation proposed by Hou et al. (2018) on the grounds of static crystallographic pictures.

As already discussed, our work has been performed using as a closed state the X-ray structure of mutant H206A resolved by Hou *et al* at 6.7 Å resolution. Recently however, a new high-resolution (3.2 Å) cryo-EM structure of mutant H206A has been published (Hou et al., 2020). This suggests the opportunity to discuss the relevance of our results in view of the new structural data. The main merit of the new structure is that it resolves the side-chains of pore-lining residues and its comparison to the closed structure (PDB: 4HKR) shows that the gating mechanism of Orai does not involve rotation of pore helices but just their translation. Our analysis of pore helix rotation was performed using a methodology that does not require knowledge of the exact position of side-chains in the experimental structures. In fact, we defined rotation angles only based on the position of *α*-Carbons. The modest main-chain RMSD (1.5 Å) between the new 7KR5 structure and the 6BBF structure employed in our study suggests that our C_*α*_-based methodology should be appropriate. Moreover, our TMD simulations were not started from the experimental structures, but from structures equilibrated for 100 ns where the position of pore helix side-chains is allowed to vary with respect to the initial crystallographic conformations. Our simulations did reveal pore helix rotation that, however, only provides a small contribution to the variation of the pore radius; this quantity mainly depends on helix displacement, in agreement with the cryo-EM structure 7KR5. It is possible that an alternative definition of the rotation angles, more dependent on the orientation of side-chains, would have led to smaller or even negligible rotation angles. However, it is also possible that the presence of antibody Fab fragments complexed with CRAC channel at the level of the TM1-TM2 loop (Hou et al., 2020), may have put helix TM1 under strain preventing its ability to rotate. Further studies will be needed to solve this problem.

Another important issue is related to the hydrophobic gating mechanism. Using an hydrophobic constriction with length similar to that of the hydrophobic region of CRAC channel, Aryal et al. (2015) observed a dewetting transition when the diameter of the construct falls below a cutoff of approximately 9 Å. When discussing the features of the 7KR5 structure, Hou et al. (2020) highlighted the fact that the diameter of CRAC hydrophobic region is 5–6 Å in the closed state (PDB: 4HKR) and 9–10 Å in the open state (PDB: 7KR5) and on this ground they predicted a hydrophobic gating mechanism. The fact that our TMD simulations showed formation and destruction of a bubble in the hydrophobic region, shows that our simulations based on the lower-resolution (6.7 Å) 6BBF H206A structure are also compatible with the higher resolution 7KR5 structure.

In short sum, our work, judiciously combining equilibrium and enhanced sampling MD simulations, highlighted the hydrophobic gating mechanism of CRAC channel and traced the allosteric coupling between the TM4 helices where the effector STIM1 binds, and the pore helices where the bubble forms and breaks. Many aspects of this mechanism require further investigation. The events following the H206 flip will have to be identified through longer or more accurate enhanced sampling simulations. Moreover, the role of a basic region in a cation channel will have to be clarified and the interplay between the electrostatic and hydrophobic gates accurately analyzed. These efforts will not only aid in the treatment of the diseases caused by mutations of this channel, but will also deepen our general understanding of hydrophobically-gated ion channels.

## 4 Materials and Methods

### 4.1 Set-Up of the Systems

The conformation of the closed state of CRAC channel was taken from the Protein Data Bank (PDB ID: 4HKR) and the missing loops 181–190 and 220–235 were modelled with the MODELLER 9.21 software (Sali and Blundell, 1993). This structure contains mutations C224S, C283T introduced to prevent non specific disulfide formation and P276R, P277R (in the hypervariable TM3-TM4 loop) introduced to produce well ordered crystals (Hou et al., 2012). In order to better compare our computational results with the experimental data in the literature, these mutations were retained. The structures of the constitutively open mutants H206A and P288L were also taken from the Protein Data Bank (PDB ID: 6BBF and 6AKI respectively). In order to prepare the systems for Targeted Molecular Dynamics simulations (Schlitter et al., 1994) that require the same number of atoms in the initial and final conformations, short N-terminal and C-terminal extensions have been added to both 6BBF (residues 144–147 and 328–334) and 6AKI (residues 144–149 and 331–334) to match the sequence length of 4HKR, using the MODELLER software (Sali and Blundell, 1993). The structures of 6BBF and 6AKI have been back-mutated to the wild type sequence (mutation A206H in 6BBF and L288P in 6AKI). In order to match the sequence of 4HKR mutations P276R and P277R have also been introduced. Protonation states have been assigned using the WHATIF server (Vriend, 1990). A number of open issues pertaining to the experimental structures of the closed state (PDB: 4HKR) and the putatively open states (PDB: 6BBF, 6AKI) is discussed in Supplementary Material section *Open issues on CRAC experimental structures*. The three channels were embedded in a lipid bilayer comprising 533 molecules of 1-palmitoyl-2-oleoyl-sn-glycero-3-phosphocholine (POPC) and solvated by 62,927 water molecules using the CHARMM Membrane Builder (Wu et al., 2014). We added 184 sodium ions and 172 chloride ions so as to neutralize the charge of the channel and reach a final concentration (NaCl) = 0.15 M. Overall, the system comprised 279,195 atoms and the simulation box had dimensions 145 × 145 × 130 Å.

### 4.2 Equilibrium MD Simulations

All simulations were performed with the NAMD 2.11b2 suite of programs (Phillips et al., 2005) using the ff15ipq force field (Debiec et al., 2016) for the protein, the Lipid17 force-field (Dickson et al., 2014) for the phospholipids and the SPC/E water model (Takemura and Kitao, 2012).

The three systems first underwent 10,000 steps of conjugate gradient minimization. During equilibration harmonic restraints were applied to nonhydrogen atoms of the protein backbone and side-chains as well as to the phospholipid heads. A harmonic restraint was also applied to the dihedral angle formed by carbons 8–11 of oleoyl acid and to the improper dihedral C_1_-C_3_-C_2_-O_2_ involving the three carbons of the glycerol unit and the hydroxyl oxygen linked to its central carbon. The equilibration was organized in 12 stages whereby the constraints were gradually released. The values of the force constants used in the 12 stages can be found in Supplementary Table S1.

The production run, following the restrained equilibration, was carried out in the isothermal isobaric ensemble for 100 ns. Pressure was kept at 1 atm by the Nosé-Hoover Langevin piston method, and the temperature was kept at 300 K by coupling to a Langevin thermostat with a damping coefficient of 1 ps^−1^. Long-range electrostatic interactions were evaluated with the smooth particle mesh Ewald algorithm. For the short-range nonbonded interactions, we used a cutoff of 12 Å with a switching function at 10.0 Å. The integration time step was 2 fs, and the bonds between hydrogen and heavy atoms were fixed to eliminate the most rapid oscillatory motions.

Pore radius profiles have been computed using the HOLE program (Smart et al., 1996). The Potential of Mean Force (PMF) of water and ions as a function of the axial position and of the distance from the pore axis was computed using equation (Im and Roux, 2002): *PMF*(*z*, *r*) = − *k*_*B*_ *T* log(*ρ*(*z*, *r*)/*ρ*_*bulk*_) where *k*_*B*_ is Boltzmann constant, *T* is the absolute temperature, *ρ*_*bulk*_ is the water or ion density in the bulk and *ρ*(*z*, *r*) is the density in position (*z*, *r*). In order to avoid a divergence of the PMF in ranges of *z* devoid of samples, we arbitrarily assign to these bins the maximal value of the free energy of the profile, *i.e.* the value of the free energy corresponding to the bin with the smallest non-zero water density. The assignment of this constant value to all the bins devoid of samples leads to the emergence of an artificially flat region near the maximum of the PMF as can be observed, e.g,*.* in Figure 3C; for this region the physical expectation is to find water molecules in the gas state. The electrostatic potential inside the pore has been computed using the PMEPot plugin (Aksimentiev and Schulten, 2005) of VMD as detailed in the *Supplementary Methods* section of the Supplementary Information. This section also provides information on our protocols to compute rotation angles, solvent accessible area and hydrophobicity profile.

### 4.3 Targeted MD Simulations

Targeted Molecular Dynamics (TMD) (Schlitter et al., 1994) is an enhanced sampling technique used to drive a system between given end states. The algorithm applies a harmonic biasing potential to the RMSD between the current conformation and the target conformation:

$$U_{TMD}=\frac{1}{2}\frac{k}{N}{\left[RMSD\left(t\right)-\rho \left(t\right)\right]}^{2}$$

where *k* is the force constant, *N* is the number of atoms where the biasing force will be applied and *ρ*(*t*) is the target RMSD that during the simulation is linearly decreased from the RMSD between initial and final structure to zero. In our case the system was steered from the final frame of the equilibrium simulation of the closed state (PDB: 4HKR) to the final frame of the equilibrium simulation of the open state (PDB: 6BBF) and *vice versa*. We applied a biasing force constant of 100,000 kcal/mol/Å^2^ and we chose a simulation lenght of 100 ns for the C → O transition and 500 ns for the O → C transition. Since the initial distance between closed and open state is 13.94 Å, this choice ensured a sufficiently slow transition with a variation of the RMSD of ∼0.14 Å/ns in the C → O and of ∼0.03 Å/ns in the O → C transition. This extremely slow protocol should allow the relaxation of the degrees of freedom that were not biased during the simulation. The good superposition between the time-evolutions of the current and target RMSD (Supplementary Figure S13) is an indication that the TMD was run slowly enough to allow the system to follow the target RMSD schedule. Finally, in order to avoid unfolding events an harmonic constraint was applied on the *α*-helical content of TM4-ext helices (force constant 50,000 kcal/mol/Å^2^).

## Data Availability

The datasets presented in this study can be found in online repositories. The names of the repository/repositories and accession number(s) can be found below: https://zenodo.org doi: 10.5281/zenodo.5497661.
