# Supplementary material for: Unveiling the Gating Mechanism of CRAC Channel: A Computational Study
Source: Front Mol Biosci. 2021 Dec 14;8:773388. doi: 10.3389/fmolb.2021.773388 (PMC8712694; doi:10.3389/fmolb.2021.773388)
Supplement: Supplementary file 1 [file Presentation1.pdf]

Supplementary Material

1 SUPPLEMENTARY RESULTS

1.1 Water and ion distribution during TMD simulations

The opening and closing TMD simulations of the CRAC channel revealed the formation or destruction of a bubble in the hydrophobic region during the final part of the simulation. In order to gain insight on the spatial distribution of water molecules and ions we computed bidimensional Potentials of Mean Force as a function of the distance r from the pore axis and the axial position z. Each PMF corresponds to a short 5 ns stretch of the TMD trajectory and we considered six such stretches at 100 ns intervals. The 5 ns stretches are so short that in each of them the RMSD form the target structure only decreases by 0.15 Å. Therefore, even if in a non-equilibrium simulation like TMD the calculation of a PMF would not be allowed, focusing on a trajectory chunk so short that the system can be considered at equilibrium restores the validity of the PMF calculation. Here we focus on the closing transition. The opening process in fact, turned out to be just the reverse of the closing.

Supporting Figure SF4 shows the water PMFs for the closing transition. In agreement with the analysis of water occupancy of the pore (Fig 6 of the main paper) the bubble forms only in the final stage of the simulation (interval 488.0-493.0 ns). It is important to note that the region devoid of water at $z \sim 20$ Å is not a hydrophobic bubble. This region corresponds to the E-ring where there is a high concentration of Na^+ ions that sterically exclude the presence of water. A similar pattern can be observed at $z \sim 0$ Å that corresponds to the K163 ring of the basic region. Here the presence of water molecules is limited by the steric exclusion due to the high number of Cl^- ions. The genuine hydrophobic bubble instead is centered at $z \sim 10$ Å and only appears in the final interval of the simulation.

It can be observed that, as the simulation proceeds, there is a narrowing of the hydrophobic region. Figure 6 (D) of the main paper shows that up to $t \sim 300$ ns the number of water molecules in the hydrophobic region remains approximately constant. As a result, the decrease in volume determines an increase of water density that becomes higher than that of the bulk. This explains why the free energy of water shifts from positive to increasingly negative (as highlighted by the color code of the free energy in the panels from 0.0-5.0 ns to 297.5-302.5 ns). In the final part of the simulation water molecules are quickly expelled from the hydrophobic region. As a result, the local density of water becomes again smaller than that of the bulk (panel 397.5-402.5 ns). After that the hydrophobic region is completely de-wetted and the bubble appears (panel 488.0-493.0 ns).

A similar analysis, shown in Supporting Figures SF5 and SF6, was performed for the chloride and sodium ions. Supporting Figure SF5 shows that for most of the simulation time chloride ions are evenly distributed in the basic region. As the simulation proceeds, there is a narrowing of the basic region. Due to Coulomb law, this determines an increase of the electrostatic potential at the level of the rings of positively charged residues. This breaks the chloride distribution in a small number of high density regions (blue islands in the PMF). What is striking however, is the fact that, despite the breakdown of the bubble and the wetting of the hydrophobic region, chloride ions remain confined in the basic region. The same applies to sodium ions (Supporting Figures SF6) that are always concentrated at the level of the E-ring.

This observation suggests that the hydrophobic and electrostatic gates might be independent so that the opening of the hydrophobic gate does not automatically result in the opening of the electrostatic gate. In

order to investigate this issue we computed electrostatic potential profiles as a function of the axial position z at different times during the simulation. The first and last profiles were computed from the equilibrium simulations of the open (PDB: 6BBF) and closed state (PDB: 4HKR). The intermediate profiles were computed from the trajectory chunks corresponding to intervals 97.5-102.5 ns, 197.5-202.5 ns, 297.5-302.5 ns, 397.5-402.5 ns.

Supporting Figure SF7 shows that the main peak of the electrostatic potential increases in two main steps at t ∼200 ns and t ∼400 ns. Both increases of the electrostatic potential are due to a narrowing of the pore. The first narrowing occurs in the 100-200 ns interval and is limited to the lower part of the pore. The second narrowing, occurring in the 300-400 ns interval, on the other hand, also involves the central and higher regions of the pore. The electrostatic potential also featureas a secondary peak corresponding to the glutamate ring. At the beginning of the simulation in this region the number of Na^+ ions is smaller than the number of glutamates and the overall charge is negative (in agreement with the initial negative potential of the secondary peak). As the simulation proceeds the E-ring is reached by a number of sodium ions that balances the number of glutamates. The increase of the secondary peak at t ∼100 ns is related to this event. The secondary peak also undergoes a significant increase around t ∼200 ns due to a transient approach of the K270 and the R276 rings. These residues later move away causing a gradual decrease of the secondary peak that returns to the level exhibited at t ∼100 ns.

We can now address the question that triggered this analysis. The barrier between the minimum separating the two peaks and the main peak is ∼22.5 $k_B T/e$ in the closed state and ∼14 $k_B T/e$ in the open state. In other words the opening of the hydrophobic gate halves the electrostatic barrier opening also the electrostatic gate. Yet, even in the open state the CRAC channel retains a significant barrier of electrostatic potential that explains the extraordinarily low conductance of this channel.

1.2 Analysis of RMSD variability

In molecular simulations it is extremely interesting to quantify the variability across runs of the physical observables of interest. This task is normally accomplished by running several repeats of each equilibrium simulation so that the time plots can be expressed a average ± SEM. In this work, due to limited availability of computational resources, we performed a single run of both the equilibriun and Targeted Molecular Dynamics simulations. In the equilibrium cases in order to have an estimate of the variability across runs, it is possible to perform a block analysis Frenkel and Smit (2002). If the trajectory is split into n_B blocks each of length τ_B, the block average of an observable A can be computed as

$$\bar{A}_B = \frac{1}{\tau_B} \int_0^{\tau_B} A(t)dt$$

Since the data blocks can be thought of as repeats of shorter trajectories, an estimate of the variability across runs can be attained by computing the variance of the block averages

$$\sigma^2(\bar{A}_B) = \frac{1}{n_B} \sum_{b=1}^{n_B} (\bar{A}_B - \langle A \rangle)^2$$

In order to apply this approach to the RMSD plots in Figure 5 of the main paper, the 100 ns equilibrium trajectory of 6AKI was split into 5 20ns-blocks. Supporting Table S3 summarizes the averages over the

whole trajectory (equal to the average of block averages) and the standard deviation of block averages. The small values of the standard deviation indicate low variability across runs.

As already noted, this approach is appropriate for equilibrium simulations where the different data blocks can be considered as equivalent repeats of a shorter trajectory. The method however, is not viable for non equilibrium simulations where the different data blocks represent simulations run under a different bias. For instance in our TMD simulations, each data block would correspond to a different value of the target RMSD, and hence to a different harmonic biasing potential. As a result, a similar analysis could not be applied to the time-plots in Figure 6-7 of the main paper.

We would like to conclude the section of RMSD analysis with two observations on the seemingly large values of RMSD appearing in Fig 5 of the main paper. First: the RMSD appearing in Figure 5 looks very large when compared to the RMSD values of a few Angstroms published in structural papers (Hou et al., 2018, 2020) comparing the open and closed conformations of CRAC channel. However, experimental comparisons usually neglect helix TM4-ext and sometimes even helix TM4b. Considering that the most important structural difference between the closed and open structure of CRAC is the extension of helices TM4b and TM4-ext, this approach leads to a significant under-estimation of the open-closed RMSD distance. Second: in Figure 5 we computed the RMSD between 6AKI, 6BBF and 4HKR. However, as discussed in the paper, there are reasons to believe that 6AKI does not represent the genuine open state, but rather an intermediate in the transition process, very different from the two most likely end states, 4HKR and 6BBF. Indeed, while the 4HKR-6AKI and 6BBF-6AKI RMSD (RMSD of backbone atoms computed using the whole TM1-TM4ext sequence, after and equilibration of 100 ns) are 37 and 38 Å respectively, the 4HKR-6BBF RMSD is only 13 Å and it reduces to approximately 5 Å if helix TM4-ext is excluded from the calculation. As a result, the conformational transition between the most likely end states occurs in a much more reasonable range of RMSD values.

1.3 Rotation of pore helices

In the main paper we described the rotation of pore helices occurring during the TMD simulations. Here, we would like to emphasize that CRAC channel does show an intrinsic tendency to rotation. Indeed, rotation of pore helices could be observed already during the 75 ns of our restrained equilibration of the system (Supporting Figure S14). During this stage of the simulation, harmonic restraints are gradually released. Supporting Figure S14 shows that as long as the restraints are strong (first 65 ns of the simulation) the rotation angles show a modest increase and the curve grows with a constant slope. As soon as the restraints are completely, or almost completely lifted, the rotation angles show an abrupt jump to a value that will be maintained during the subsequent 100 ns of the unrestrained equilibrium run.

In Supporting Table S4 we show the average rotation angles in the four critical regions of the pore computed: (i) during the restrained equilibration run of the closed structure (4HKR), (ii) during the 100 ns of the C$\longrightarrow$O TMD simulation, (iii) in the comparison of the experimental structures of the closed (4HKR) and open (6BBF) states of CRAC channel. Supporting Table S4 shows that the pore helices of the experimental structure of the open state (6BBF) appear to be rotated with respect to the resting structure (4HKR). Moreover, the sum of the rotation angles measured during the restrained equilibration and during the TMD simulation is approximately equal to the rotation angles measured during the comparison of the experimental structures. This is a clear evidence that the rotation angles measured during the TMD runs are not an artifact introduced by the Targeted Molecular Dynamics algorithm.

2 SUPPLEMENTARY METHODS

2.1 Open issues on CRAC experimental structures

The quality of a molecular simulation work like ours, critically depends on the accuracy of the input structures. It is thus important to briefly summarize the ongoing debate in the literature on a number of technical issues affecting the closed and open structures of CRAC channel. A key element of the closed conformation (4HKR) is represented by the three anti-parallel coiled-coils between the TM4-ext helices of neighbouring subunits. Some authors (Zhou et al., 2019) however, have argued that these structural elements might be an artifact due to the fact that the crystal lattice was stabilized through a Zn^{2+}-mediated trimeric association of His-pairs at the C-terminus of each TM4-ext (Hou et al., 2012). The uncertainty on the closed state structure has been dissipated through extensive experimental testing. For instance, it has been shown (Muik et al., 2008) that mutation of I316 or L319 that mediate the hydrophobic interactions stabilizing the coiled-coil motif, completely blocks STIM1-binding and channel activation. Moreover, it was also shown (Tirado-Lee et al., 2015) that cross-linking of TM4-ext helices prevents channel activation by STIM1.

The putative open conformations of CRAC channel, instead, are still under scrutiny. In the crystal structures of both the H206A mutant (PDB: 6BBF) (Hou et al., 2018) and the P288L mutant (PDB: 6AKI) (Liu et al., 2019) the CRAC channel forms dimers stabilized by 12 inter-molecular anti-parallel coiled-coils between TM4-ext helices. These interactions, analogous to the pairings of M4-ext helices of neighbouring subunits in the quiescent state, are a likely artifact of the crystallization conditions. Yet, these interactions are strong enough to unlatch the TM3-TM4 interactions, creating the so-called *unlatched-closed* conformations. As discussed more in detail in the *Discussion* section of the main paper, a dynamical perspective, also supported by the results of our MD simulations, shows that unlatched-closed conformations can be thought of as intermediates of the gating process, and the existence of these hybrid conformations does not undermine the importance of TM4-ext un-latching for the dissolution of the bubble in the hydrophobic region. Here we would like to add that the structural similarity (mainchain RMSD of 1.5 Å) between the low-resolution X-ray structure of H206A (PDB: 6BBF) (Hou et al., 2018) and the high-resolution cryo-EM structure of the same mutant (PDB: 7KR5) (Hou et al., 2020), shows that the un-latched conformation was attained using completely different experimental conditions. Moreover, our equilibrium simulation of 6BBF shows that the un-latched conformation remains stable for the whole 100 ns length of our run, which would probably not be true for a purely artifactual conformation. Yet, the debate is still open (Zhou et al., 2019, 2016; Butorac et al., 2019) and an ultimate answer will be attained only when the structure of the STIM1-CRAC complex is finally resolved.

2.2 Electrostatic potential profile

The electrostatic potential inside the pore has been computed using the PMEPot plugin (Aksimentiev and Schulten, 2005) of VMD. The plugin computes the potential using the Particle Mesh Ewald algorithm, the same used to compute long-range electrostatic interactions in Molecular Dynamics simulations. In this algorithm a point charge q_i is spread in space by means of a Gaussian distribution $\rho_i(\mathbf{r})$. The algorithm then solves the Poisson equation

$$\nabla^2\phi(\mathbf{r}) = -\frac{4\pi}{\epsilon}\sum_i \rho_i(\mathbf{r})$$

The algorithm discretizes the calculation of the electrostatic potential ϕ embedding the system in a 3D-grid with grid spacing slightly smaller than 1.0 Å and computing the electrostatic potential only at grid points. The grid density determines the accuracy of the resulting potential. To determine the mean electrostatic potential, instantaneous electrostatic potentials $\phi_i(\mathbf{r})$ computed at each frame, were averaged over the entire MD trajectory. The electrostatic potential profile along the axis of the pore was then attained by averaging the potential $\phi_i(\mathbf{r})$ over the x and y coordinates of the membrane plane.

2.3 Pore helix rotation angles

The rotation of pore helix TM1 around its axis was computed as follows. In a perfect helical geometry the axial coordinate z of residue n can be computed as $z(n) = n\Delta z$ so that

$$\frac{z(n) + z(n+4)}{2} = z(n+2)$$

This means that the vector connecting the center of mass of C_α atoms n and $n+4$ with the C_α atom $n+2$ is orthogonal to the helix axis. The angle α between this vector, $\vec{v}^{rot}(t)$ and its counterpart $\vec{v}^{rot}(0)$ in the initial conformation defines the rotation angle. To account for local deformations of the helical geometry that might undermine the orthogonality of $\vec{v}^{rot}$ to the axis, the calculation was performed using the component of $\vec{v}^{rot}$ orthogonal to the axis:

$$\alpha = \arccos\left(\frac{\vec{v}^{rot}_\perp(t) \cdot \vec{v}^{rot}_\perp(0)}{|\vec{v}^{rot}_\perp(t)| \cdot |\vec{v}^{rot}_\perp(0)|}\right)$$

In order to identify possible *twisting* events we computed four different rotation angles at different levels along the pore: (i) glutamate ring, (ii) hydrophobic region, (iii) upper basic region, (iv) lower basic region. A further layer of complexity is related to the fact that pore helices of CRAC channel are not straight but bent, so that there are two axes, one in the upper and one in the lower part of the helix. This is why vector $\vec{v}^{rot}$ must always be projected on the appropriate axis corresponding to the region where the measured angle is located.

Supporting Table S2 lists the residues that were used to compute the generating vectors $\vec{v}^{rot}$ of the rotation angles. The three columns of the Table correspond to the three residues n, $n+4$ and $n+2$. For each pore helix we considered four triplets of residues corresponding to the angles at the level of the glutamate ring, hydrophobic region, upper and lower basic region. The upper and lower axis of each TM1 helix is defined by two quartets of residues whose initial elements are: 176, 169, 154, 147. In this list the first two numbers represent the initial elements of the upper and lower quartet of the upper axis, while the third and fourth numbers represent the initial elements of the upper and lower quartet of the lower axis.

2.4 Solvent Accessible Surface Area

The Solvent Accessible Surface Area (SASA) of the pore wall has been computed using the NACCESS code (Hubbard and Thornton, 1993). This program is an implementation of the algorithm by Lee and Richards (Lee and Richards, 1971) whereby a spherical probe having the radius of a water molecule (1.4 Å) is rolled on the ensemble of van der Waals spheres representing the atoms of the protein. The Solvent Accessible Surface Area is then the surface spanned by the center of the rolling sphere. In order to compute the SASA of the pore wall we need to sum the contributions of the side-chains leaning into the pore lumen. These side chains are identified using simple geometric criteria. The first requirement is that the distance

r_{COM} of the center of mass of the side chain from the pore axis at a given height z does not exceed the pore radius r_{pore} (computed using the HOLE software) plus a tolerance r_{thr}=7.5 Å.

$$r_{COM} < r_{pore} + r_{thr}$$

In order to make sure that the side-chain points inwards towards the centre of the pore we also require that the distance r_{COM} of the side-chain from the pore axis be smaller than the distance r_{CA} from the pore axis of the α-carbon of the same residue:

$$r_{COM} < r_{CA}$$

2.5 Hydrophobicity profile

The hydrophobicity profile was computed using an in-house developed code based on the same algorithm implemented in the CHAP program (Klesse et al., 2019) of the Sansom group. The program first identifies the side chains lining the pore wall employing the same geometric criteria also used for the SASA calculation. Each pore-lining residue is then assigned a hydrophobicity value taken from the Wimley-White hydrophobicity scale (Wimley and White, 1996). The Wimley-White hydrophobicities are just free energies of transfer from the bilayer/water interface to the bulk water of a peptide whose central residue is the amino-acid of interest. As a result, hydrophobic amino-acids are assigned positive hydrophobicities ($\Delta G > 0$) while hydrophilic amino-acids will be assigned negative hydrophobicities ($\Delta G < 0$). In order to attain a smooth curve, the hydrophobicity profile is computed as a kernel-weighted average of hydrophobicities H_i:

$$H(z) = \frac{\sum_i K_\lambda(z, z_i) H_i}{\sum_i K_\lambda(z, z_i)}$$

where the Gaussian kernel

$$K_\lambda(z, z_i) = \exp\left(-\frac{(z - z_i)^2}{2\lambda^2}\right)$$

has the function to spread in space along the z-axis the hydrophobicity H_i of residue i whose side-chain is centered in position z_i. Following Ref (Klesse et al., 2019) for each Gaussian we chose a width λ=1.5 Å.

3 SUPPLEMENTARY TABLES AND FIGURES

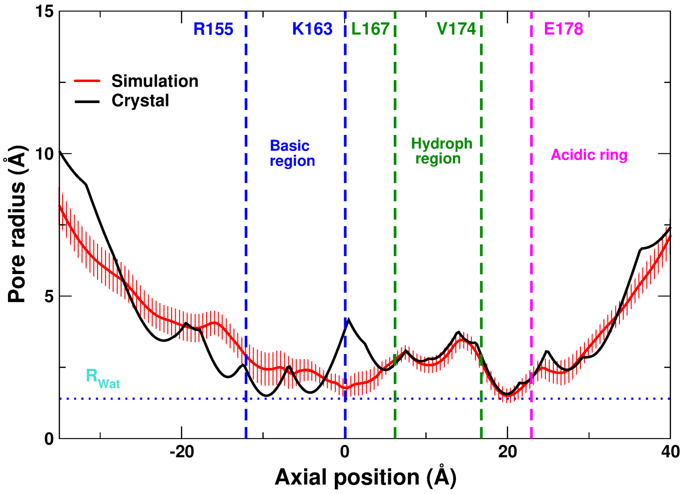

Figure S1. Radius profile of the closed state of CRAC channel (PDB ID: 4HKR). The black curve represents the radius profile of the crystal structure while the red curve is the radius profile averaged over all frames of the 100 ns equilibrium simulation. Error bars correspond to the standard deviation of the pore radius during the simulation. The radius profile is compared with the radius of a single water molecule (1.35 Å; dotted blue curve). The dashed lines mark the boundaries of the basic region (average axial position of R155 and K163 rings; blue dashed lines), hydrophobic region (average axial position of L167 and V174 rings; dashed green lines) as well as the position of the glutamate ring (magenta line).

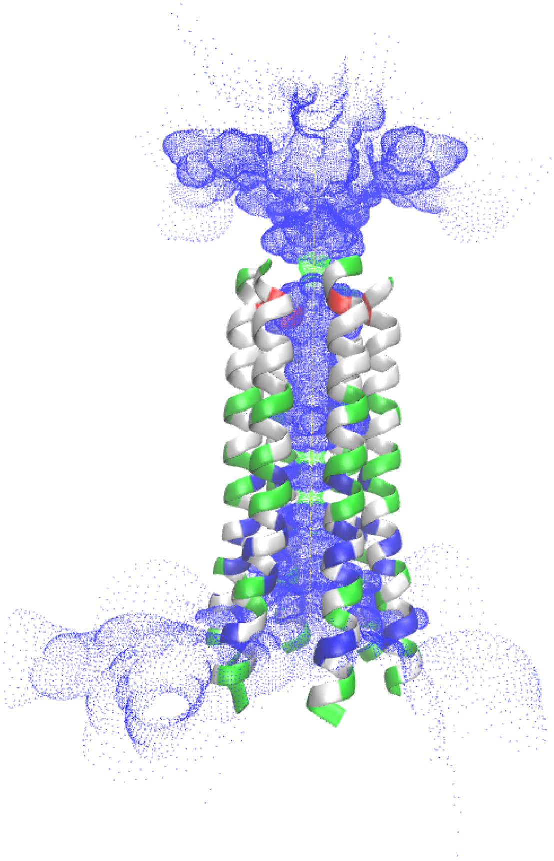

Figure S2. Volume filling representation of the closed state of CRAC channel (PDB 4HKR). The color code of the pore volume is as follows: green regions can accommodate at most a single water molecule; blue regions can host two or more water molecules. Pore helices are colored according to the residue type: positively charged residues are painted in blue, negatively charged residues in red, polar but neutral residues in green and hydrophobic residues in white.

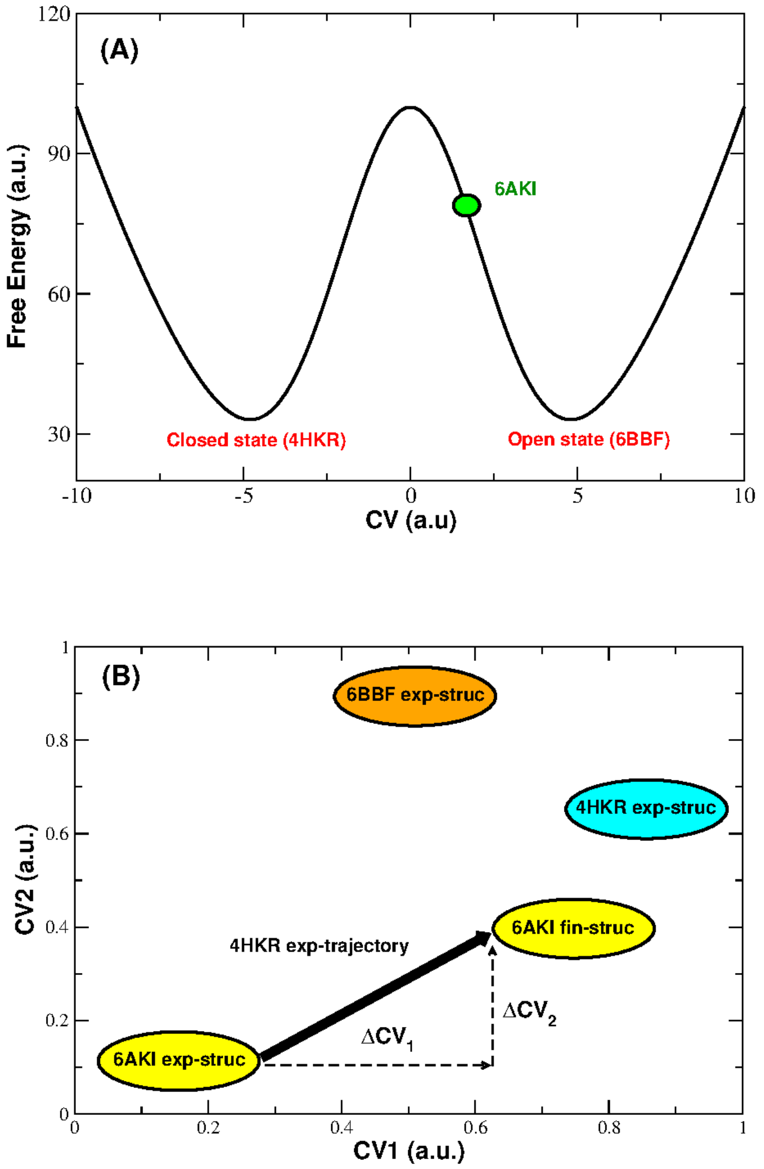

Figure S3. Cartoon representation of the free energy landscape of the gating transition of CRAC channel. Panel **(A)**: free energy is assumed to be function of a single collective variable (CV). The closed (4HKR) and open state (6BBF) correspond to two minima separated by a barrier. Due to its structural instability 6AKI is predicted to be located somewhere along the barrier. In this scenario if 6AKI becomes more similar to 4HKR, it should automatically become more different from 6BBF in disagreement with the RMSD plots shown in Fig 5 of the main paper. Panel **(B)**: a more realistic scenario where free energy is function of two collective variables CV1 and CV2. In this landscape 6AKI can simultaneously increase its structural similarity with 4HKR and 6BBF in agreement with our RMSD analysis.

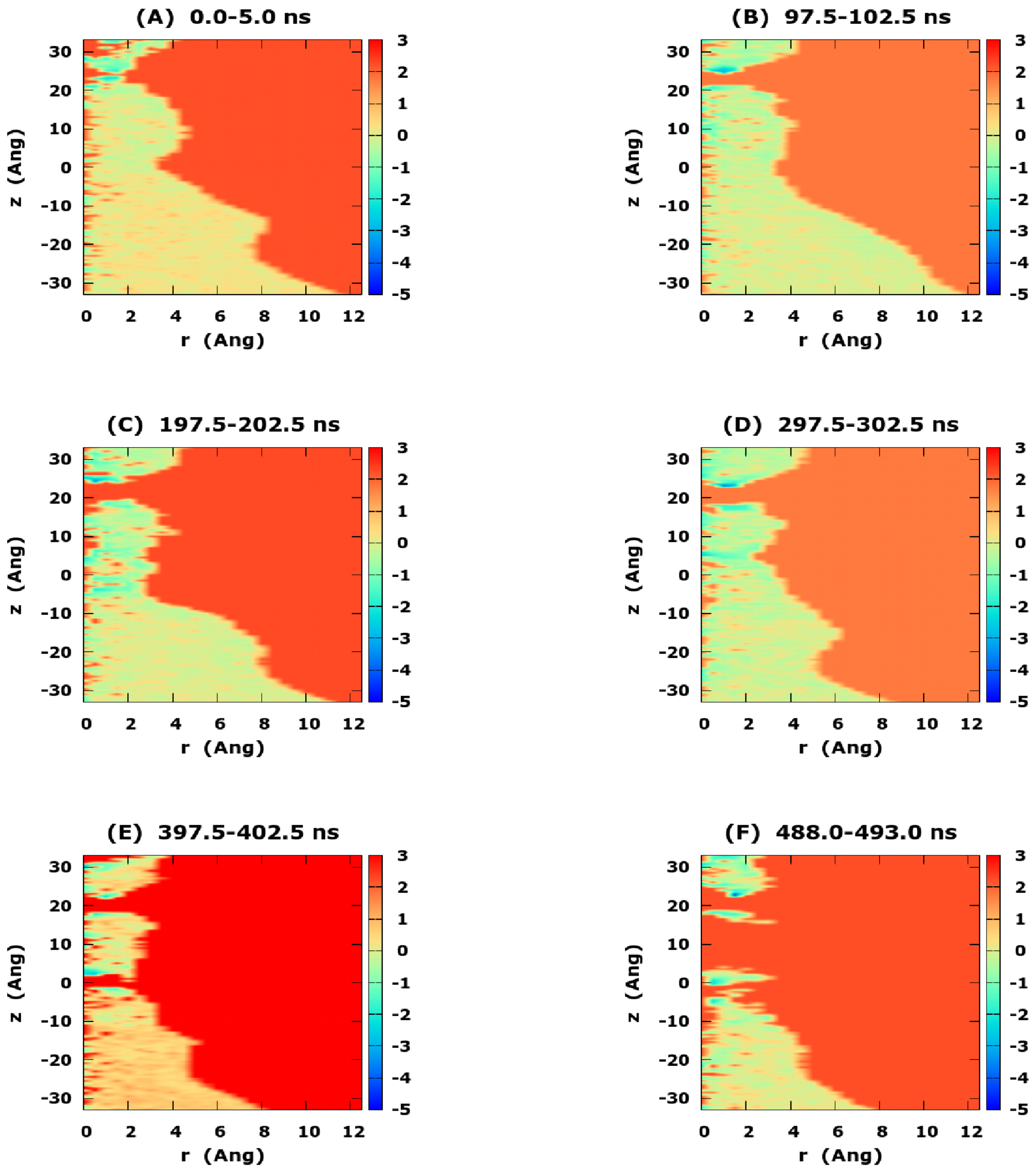

Figure S4. Potential of Mean Force of water as a function of the distance r from pore axis and the axial position z in the closing TMD simulation. Panels **(A)** to **(F)** correspond to time intervals 0.0-5.0 ns, 97.5-102.5 ns, 197.5-202.5 ns, 297.5-302.5 ns, 397.5-402.5 ns, 488.0-493.0 ns. The free energy scale is expressed in kcal/mol.

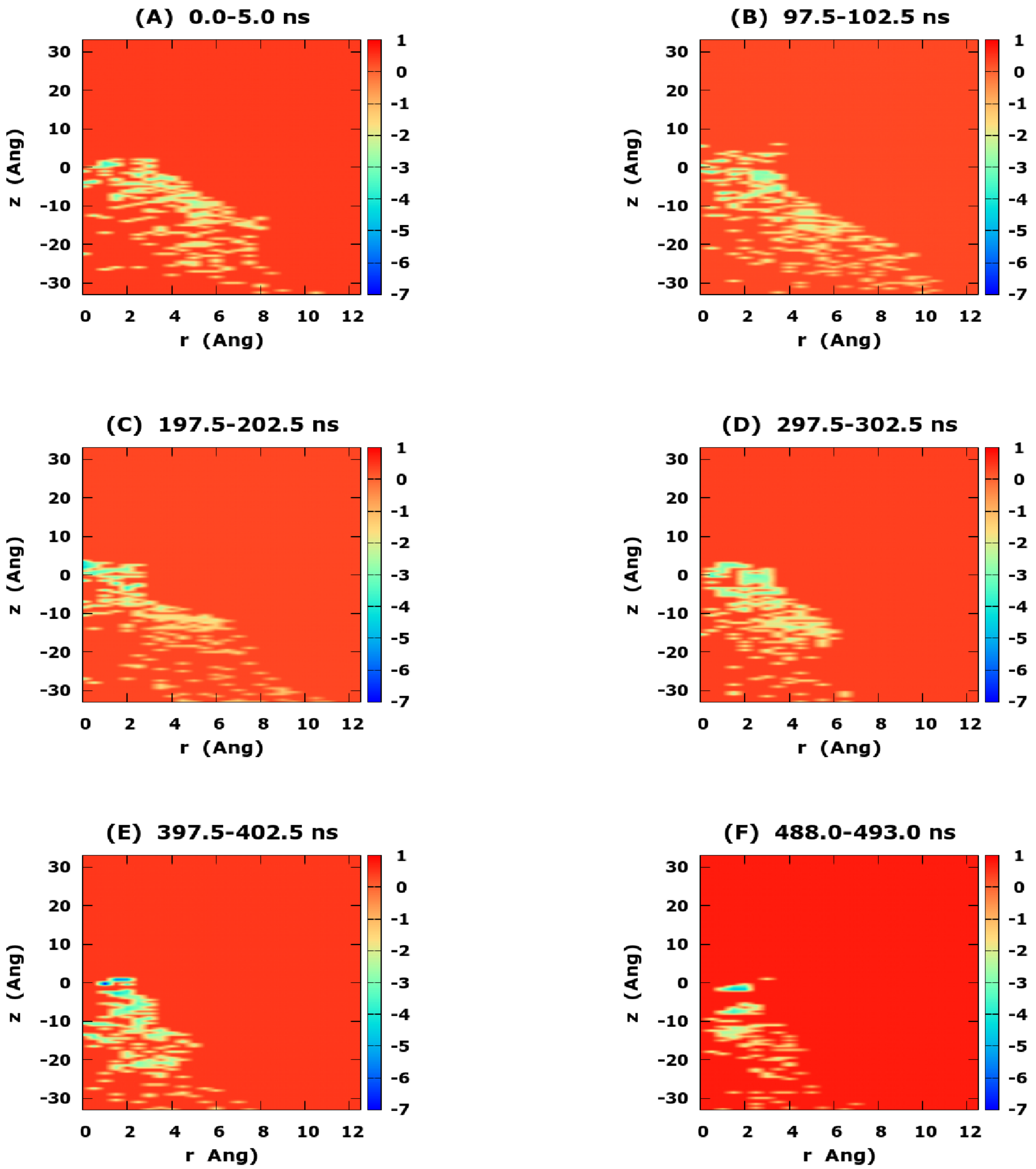

Figure S5. Potential of Mean Force of chloride ions as a function of the distance r from pore axis and the axial position z in the closing TMD simulation. Panels **(A)** to **(F)** correspond to time intervals 0.0-5.0 ns, 97.5-102.5 ns, 197.5-202.5 ns, 297.5-302.5 ns, 397.5-402.5 ns, 488.0-493.0 ns. The free energy scale is expressed in kcal/mol.

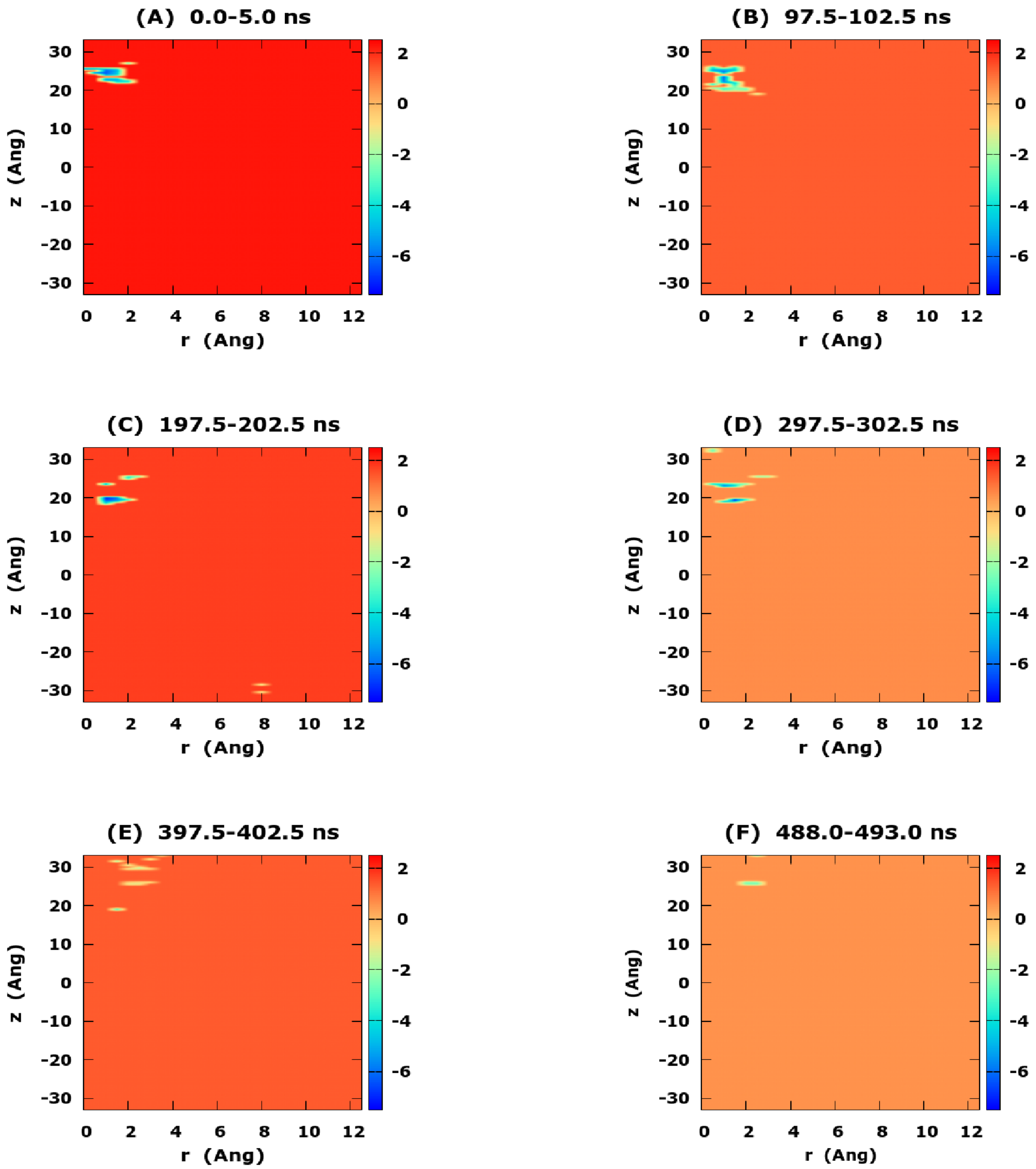

Figure S6. Potential of Mean Force of sodium ions as a function of the distance r from pore axis and the axial position z in the closing TMD simulation. Panels **(A)** to **(F)** correspond to time intervals 0.0-5.0 ns, 97.5-102.5 ns, 197.5-202.5 ns, 297.5-302.5 ns, 397.5-402.5 ns, 488.0-493.0 ns. The free energy scale is expressed in kcal/mol.

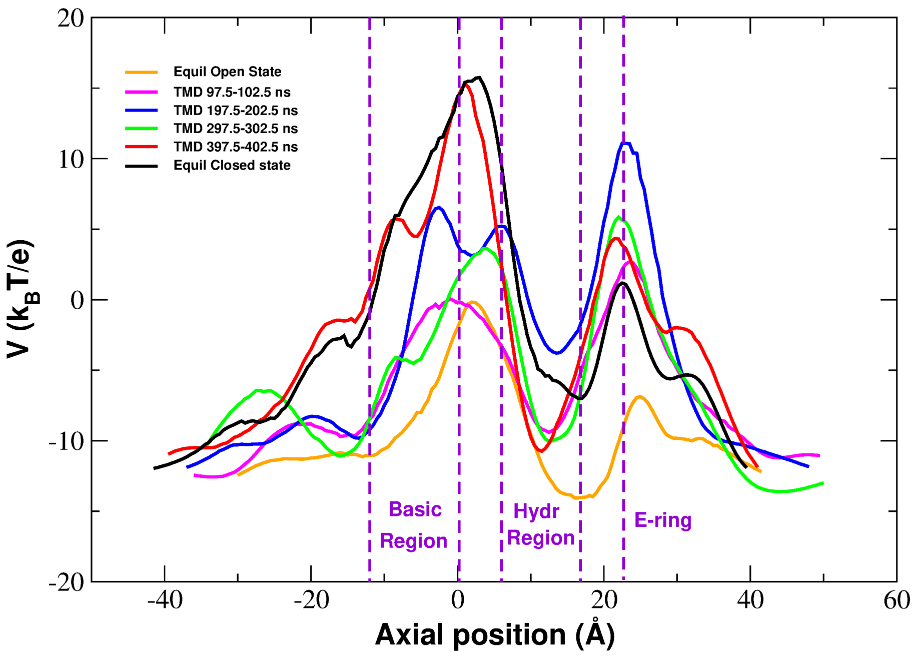

Figure S7. Evolution of the electrostatic potential profile during the closing transition of the CRAC channel. Potentials were computed during the equilibrium simulations of the open (PDB: 6BBF; orange curve) and closed states (PDB: 4HKR; black curve) as well as in four 5 ns intervals taken during the O→C TMD simulation (97.5-102.5 ns: magenta curve; 197.5-202.5 ns: blue curve; 297.5-302.5 ns: green curve; 397.5-402.5 ns: red curve). The dashed lines mark the boundaries of the basic and hydrophobic region as well as the position of the E-ring.

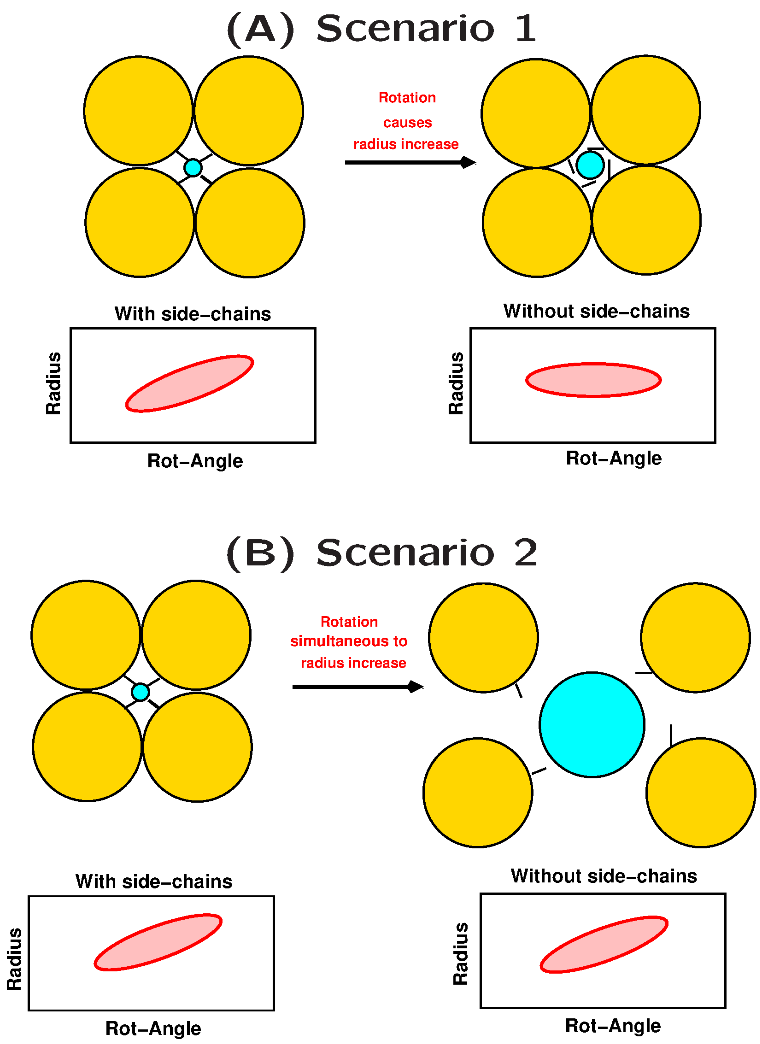

Figure S8. A computational strategy to check for the influence of pore helix rotation on pore radius. Panel **(A)** depicts a scenario wherby the rotation of pore helices, displacing the bulky side chains of hydrophobic residues does cause the increase of pore radius. A signature of this event can be attained by computing a Potential of Mean Force as a function of rotation angle and pore radius. The calculation is repeated accounting for the side chains in the calculation of pore radius and computing the pore radius using the backbone atoms only. In the former case the low free energy region of the PMF is expected to be slanted, in the latter it will be flat. Panel **(B)** illustrates a second scenario where rotation of pore helices and increase of pore radius are simultaneous events with no causal link between them. In this case the increase in pore radius is due to the displacement rather than the rotation of pore helices. In this case either computing the PMF including or excluding side chains, the low free energy region will be tilted.

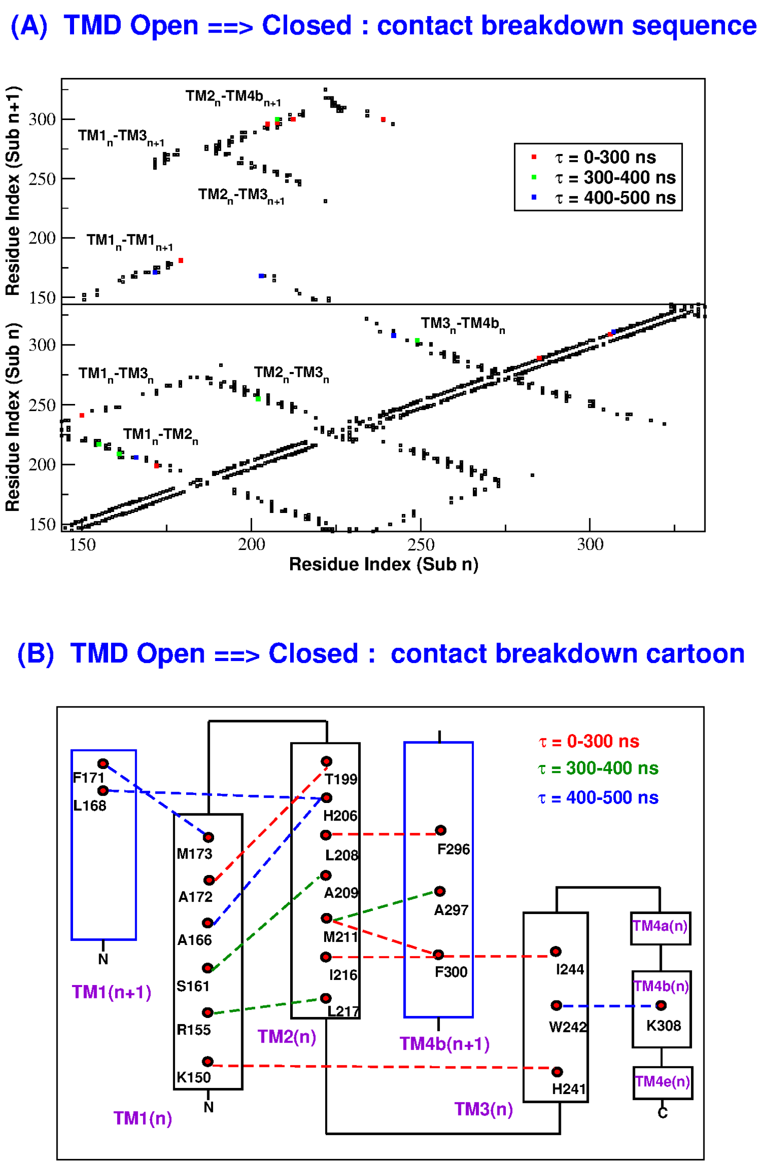

Figure S9. Sequence of contact breakdown in the TMD closing simulation. Panel **(A)**: contact map. Black squares are contacts present in the open state and not broken during the simulation. Coloured squares are contacts broken at different times τ during the simulation. The lower part of the map shows intra-subunit contacts, the upper part contacts between subunits n and $n + 1$. Panel **(B)**: cartoon representation of contact breakdown. Broken contacts are represented by dashed lines with color-coded breakdown time τ.

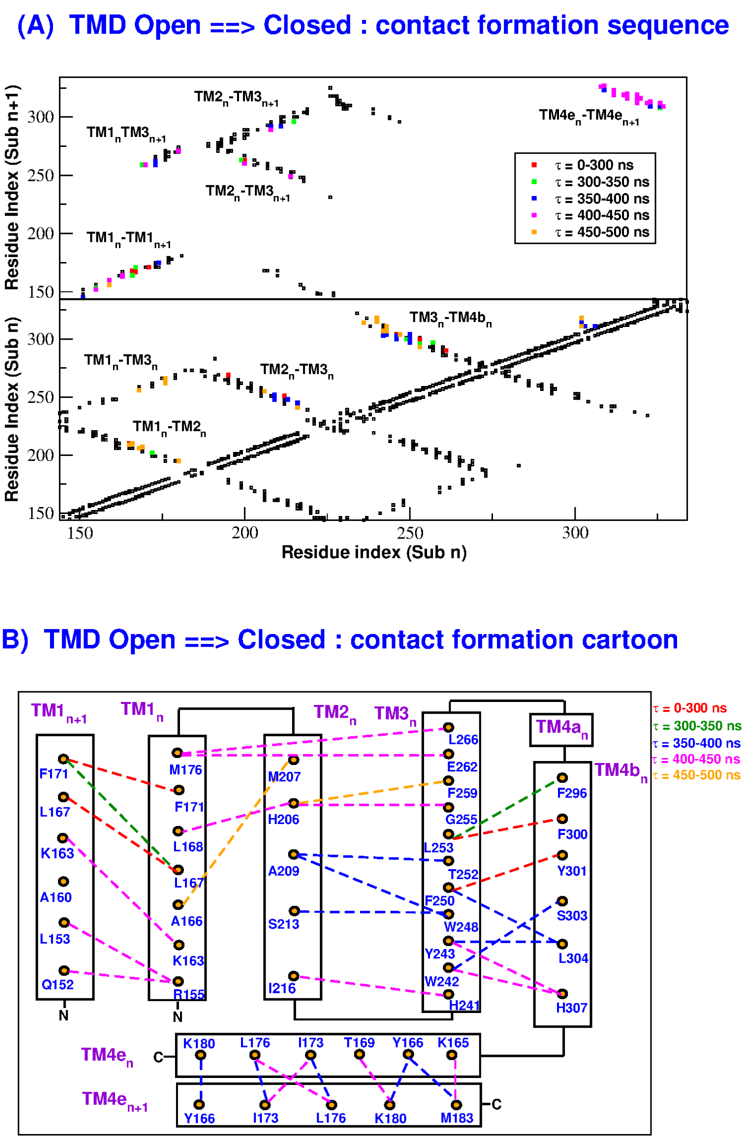

Figure S10. Sequence of contact formation in the TMD closing simulation. Panel (**A**): contact map. Black squares are contacts present in the open state and not broken during the simulation. Coloured squares are contacts formed at different times τ during the simulation. The lower part of the map shows intra-subunit contacts, the upper part contacts between subunits n and $n+1$. Panel (**B**): cartoon representation of contact formation. Formed contacts are represented by dashed lines with color-coded formation time τ.

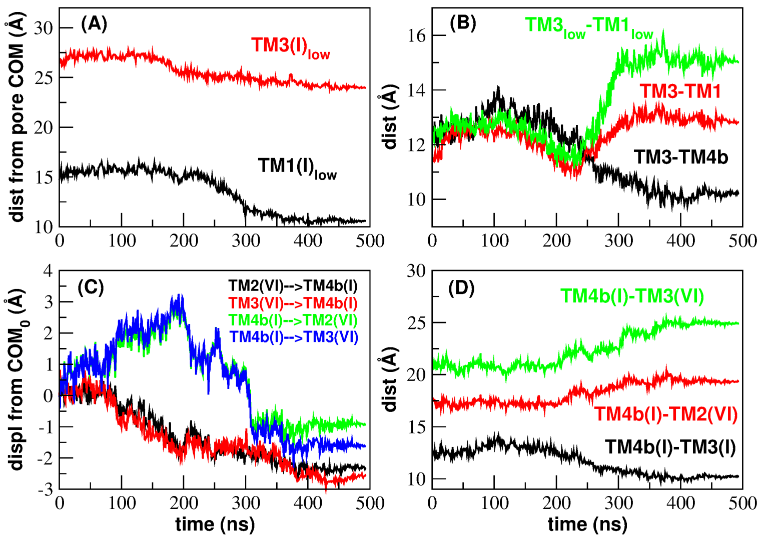

Figure S11. Inter-helical distances involving helices TM3 and TM4b of Subunit I during the closing TMD simulation. Panel **(A)**: distance of the lower part of helices TM1 and TM3 from the Center of Mass (COM) of the pore. Panel **(B)**: the decrease of the distance TM3-TM4b is paralleled by an increase of the TM3-TM1 distance (more pronounced if considering only the lower part of the two helices). Panel **(C)**: displacement of the center of mass of helices TM4b(I), TM2(VI), TM3(VI) from its initial position. The displacement vector is projected such that a positive displacement represents an approach while a negative displacement a distancing between the helices. Panel **(D)**: the decrease of the distance TM3(I)-TM4b(I) is paralleled by an increase of the TM4b(I)-TM3(VI) and TM4b(I)-TM2(VI) distances.

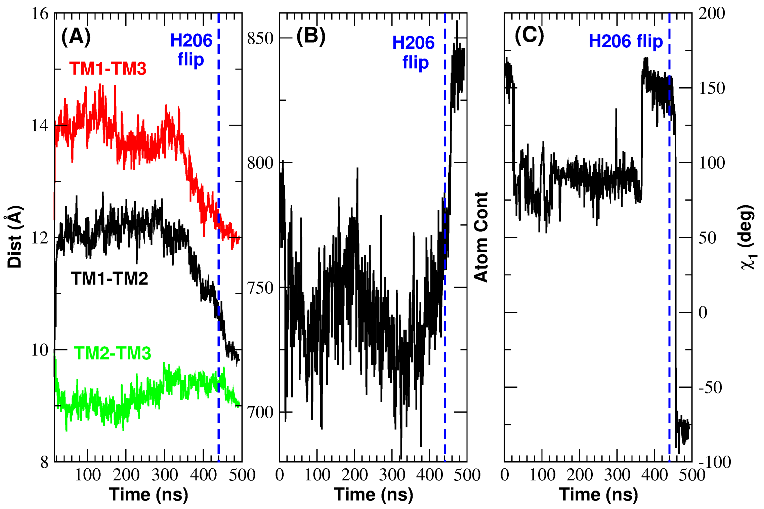

Figure S12. Rearrangement of H206 during the closing TMD simulation. Panel **(A)**: inter-helical distances TM1-TM2 (black curve), TM1-TM3 (red curve) and TM2-TM3 (green curve). Panel **(B)**: number of atomic contacts of H206 as defined by a distance cutoff of 7.5 Å. Panel **(C)**: dihedral angle χ_1 of H206.

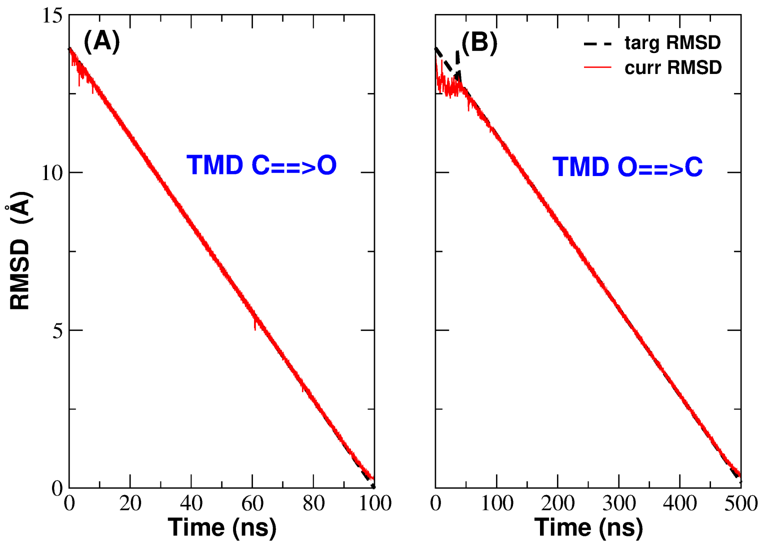

Figure S13. Actual (red curve) and target (black curve) RMSD profiles during the 100 ns C⟶O TMD simulation (Panel (**A**)) and the 500 ns O→C TMD simulation (Panel (**B**)).

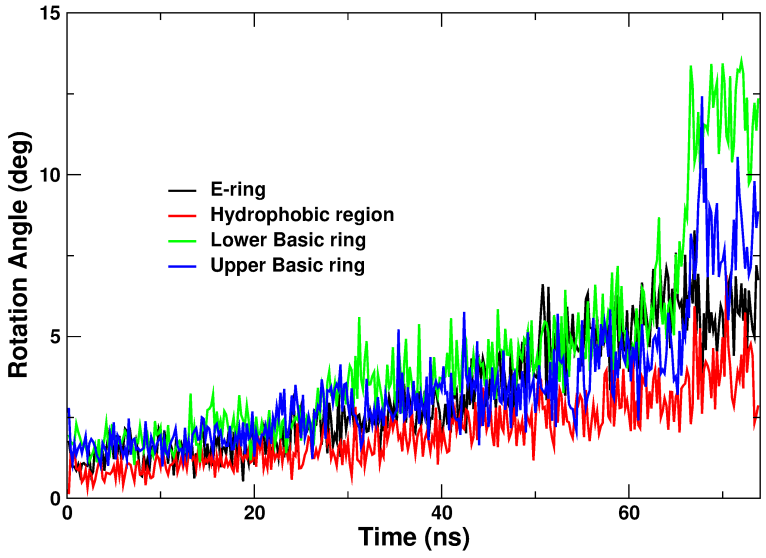

Figure S14. Rotation of pore helices during the 75 ns of the restrained equilibration of the closed state (PDB: 4HKR) of CRAC channel. The curves are averaged over the six subunits of the channel. The color code is the following. Black curve: E-ring, red curve: hydrophobic region, green curve: lower basic region, blue curve: upper basic region.

Stage	Ens	BB	LH	DIH	δt	dcdfreq	Steps
Eq0	NVT	20	50	950	1.0	5000	10K+50K
Eq1	NPT	20	50	950	1.0	5000	4.5×10^6
Eq2	NPT	16	40	900	1.0	5000	4.5×10^6
Eq3	NPT	12	30	800	1.0	5000	4.5×10^6
Eq4	NPT	8	20	700	1.0	5000	4.5×10^6
Eq5	NPT	6	10	600	1.0	5000	4.0×10^6
Eq6	NPT	4	5	500	1.0	5000	4.0×10^6
Eq7	NPT	3	5	200	2.0	5000	4.0×10^6
Eq8	NPT	2	2	100	2.0	5000	4.5×10^6
Eq9	NPT	1	1	50	2.0	5000	4.0×10^6
Eq10	NPT	0.5	0.5	25	2.0	5000	4.0×10^6
Eq11	NPT	0.25	0.25	10	2.0	5000	4.0×10^6
Eq12	NPT	0.0	0.0	0.0	2.0	5000	4.0×10^6
Eq-free	NPT	0.0	0.0	0.0	2.0	5000	5.0×10^7

Table S1. Constrained equilibration protocol. For each stage of the simulation the Table reports the statistical ensemble (Ens) and the force constants (in kcal/(mol·Å^2)) of the harmonic restraints that have been applied to protein backbone (BB), the polar heads of the phospholipids (LH) and to some selected dihedral angles of the lipids (DIH). The Table also shows the time-step (in fs), the sampling frequency of the trajectory (dcdfreq; number of steps) and the total number of steps of the simulation.

Rot-angle	res-n	res-$n+4$	res-$n+2$
E-ring	176	180	178
Hydrophob Reg	169	173	171
Upp Basic Reg	154	158	156
Low Basic Reg	147	151	149

Table S2. List of residues defining the generating vectors of the four rotation angle computed in the pore helices.

Observable	average [Å]	St. Dev. [Å]
RMSD$_{all}$ 6AKI-6BBF	37.36	0.29
RMSD$_{TM4}$ 6AKI-6BBF	49.16	0.55
RMSD$_{PD}$ 6AKI-6BBF	19.71	0.07
RMSD$_{all}$ 6AKI-4HKR	35.29	0.65
RMSD$_{TM4}$ 6AKI-4HKR	46.44	1.44
RMSD$_{PD}$ 6AKI-4HKR	16.63	0.10

Table S3. Block averages and standard deviations of the RMSD between 6AKI and 6BBF or 4HKR (computed as detailed in Supporting Material section *Analysis of RMSD variability*). The equilibrium simulation of 6AKI has been split into 5 20ns-blocks. The RMSD was computed aligning the current and reference structure using all backbone atoms while the RMSD was computed using all backbone atoms (RMSD$_{all}$), the backbone atoms of helices TM4 (RMSD$_{TM4}$) or the backbone atoms of the pore domain TM1 (RMSD$_{PD}$).

Pore region	Restr. Equil. [deg]	TMD C→O [deg]	Exper. Sruc. [deg]
E-ring	3.24 (2.95)	5.54 (4.24)	6.43 (0.88)
Hydrophob. Reg.	2.06 (1.67)	7.35 (5.53)	10.63 (0.73)
Upp. Basic Reg.	3.40 (3.10)	20.49 (15.45)	23.30 (0.85)
Low. Basic Reg.	4.36 (4.09)	21.90 (20.03)	20.62 (2.23)

Table S4. Average rotation angles of pore helices computed at different levels along the pore axis. Averages and standard deviations (in brackets) are computed over the whole simulation time and over the six subunits of the protein. Column 2 refers to the equilibration run of the closed state 4HKR with gradually decreasing harmonic constraints. Column 3 refers to the 100 ns TMD simulation from the closed (4HKR) to the open state (6BBF). Column 4 shows rotation angles measured during the comparison of the experimental structures of the open (6BBF) and closed state (4HKR) of CRAC channel. In this case averages and standard deviations are computed over the six subunits. All rotation angles are expressed in degrees.

REFERENCES

Aksimentiev, A. and Schulten, K. (2005). Imaging alpha-hemolysin with molecular dynamics: Ionic conductance, osmotic permeability and the electrostatic potential map. *Biophysical Journal* 88, 3745–3761

Butorac, C., Muik, M., Derler, I., Stadlbauer, M., Lunz, V., and Krizova, e. a., A. (2019). A novel stim1-orai1 gating interface essential for crac channel activation. *Cell Calcium* 79, 57–67. doi:10.1016/j.ceca.2019.02.009

Frenkel, D. and Smit, B. (2002). *Understanding molecular simulation* (San Diego, San Francisco, New York, Boston, London, Sydney, Tokyo: Academic Press, a Divistion of Harcours Inc)

Hou, X., Burstein, S., and Long, S. (2018). Structures reveal opening of the store-operated calcium channel orai. *eLife* 7, e36758. doi:10.7554/eLife.36758

Hou, X., Outhwaite, I., Pedi, L., and Long, S. (2020). Cryo-em structure of the calcium release-activated calcium channel orai in an open conformation. *eLife* 9, e62772. doi:10.7554/eLife.62772

Hou, X., Pedi, L., Diver, M., and Long, S. (2012). Crystal structure of the calcium release-activated calcium channel orai. *Science* 338, 1308–1313. doi:10.1126/science.1228757

Hubbard, S. and Thornton, J. (1993). *'NACCESS', Computer Program* (University College London: Department of Biochemistry and Molecular Biology)

Klesse, G., Shanlin, R., Sansom, M., and Tucker, S. (2019). Chap: a versatile tool for the structural and functional annotation of ion channel pores. *J. Mol. Biol.* 431, 3353–3365

Lee, B. and Richards, F. (1971). The interpretation of protein structures: estimation of static accessibility. *J. Mol. Biol.* 55, 379–400

Liu, X., Wu, G., Yu, Y., Chen, X., Ji, R., Lu, J., et al. (2019). Molecular understanding of calcium permeation through the open orai channel. *PLoS Biology* 17, e3000096. doi:10.1371/journal.pbio.3000096

Muik, M., Frischauf, I., Derler, I., Fahmer, M., Bergsmann, J., and Eder, e. a., P. (2008). Dynamic coupling of the putative coiled-coil domain of orai1 channel activation. *J. Biol. Chem.* 283, 8014–8022. doi:10.1074/jbc.M708898200

Tirado-Lee, L., Yamashita, M., and Prakriya, M. (2015). Conformational changes in the orai1 c-terminus evoked by stim1 binding. *PLoS ONE* 10, e0128622. doi:10.1371/journal.pone.0128622

Wimley, W. and White, S. (1996). Experimentally determined hydrophobicity scale for proteins at membrane interfaces. *Nature Struct. Biol.* 3, 842–848

Zhou, Y., Cai, X., Loktionova, N., Wang, X., Nwokonko, R., and Wang, e. a., X. (2016). The stim1-binding site nexus remotely controls orai1 channel gating. *Nat. Commun.* 7, 13725. doi:10.1038/ncomms13725

Zhou, Y., Nwokonko, R., Baraniak, J. J., Trebak, M., Lee, K., and Gill, D. (2019). The remote allosteric control of orai channel gating. *PLoS Biol.* 17, e3000413. doi:10.1371/journal.pbio.3000413
